# Towards Long Term Cultivation of *Drosophila* Wing Imaginal Discs *In Vitro*

**DOI:** 10.1371/journal.pone.0107333

**Published:** 2014-09-09

**Authors:** Björn Handke, János Szabad, Peter V. Lidsky, Ernst Hafen, Christian F. Lehner

**Affiliations:** 1 Institute of Molecular Life Sciences (IMLS), University of Zurich, Zurich, Switzerland; 2 Department of Biology, Faculty of Medicine, University of Szeged, Szeged, Hungary; 3 Department of Biology, Institute of Molecular Systems Biology (IMSB), ETH Zurich, Zurich, Switzerland; University of Dayton, United States of America

## Abstract

The wing imaginal disc of *Drosophila melanogaster* is a prominent experimental system for research on control of cell growth, proliferation and death, as well as on pattern formation and morphogenesis during organogenesis. The precise genetic methodology applicable in this system has facilitated conceptual advances of fundamental importance for developmental biology. Experimental accessibility and versatility would gain further if long term development of wing imaginal discs could be studied also in vitro. For example, culture systems would allow live imaging with maximal temporal and spatial resolution. However, as clearly demonstrated here, standard culture methods result in a rapid cell proliferation arrest within hours of cultivation of dissected wing imaginal discs. Analysis with established markers for cells in S- and M phase, as well as with *RGB* cell cycle tracker, a novel reporter transgene, revealed that in vitro cultivation interferes with cell cycle progression throughout interphase and not just exclusively during G1. Moreover, quantification of EGFP expression from an inducible transgene revealed rapid adverse effects of disc culture on basic cellular functions beyond cell cycle progression. Disc transplantation experiments confirmed that these detrimental consequences do not reflect fatal damage of imaginal discs during isolation, arguing clearly for a medium insufficiency. Alternative culture media were evaluated, including hemolymph, which surrounds imaginal discs during growth in situ. But isolated larval hemolymph was found to be even less adequate than current culture media, presumably as a result of conversion processes during hemolymph isolation or disc culture. The significance of prominent growth-regulating pathways during disc culture was analyzed, as well as effects of insulin and disc co-culture with larval tissues as potential sources of endocrine factors. Based on our analyses, we developed a culture protocol that prolongs cell proliferation in cultured discs.

## Introduction

In the dipteran fly *Drosophila melanogaster*, wings develop during the larval and pupal stages from a pair of imaginal discs that are set aside during embryogenesis as small pockets of around 40 epithelial cells [1], [2]. Research on *Drosophila* wing development has provided pioneering insight into mechanisms controlling pattern formation (including morphogen activity and compartment boundaries), cell interactions, growth and proliferation [3]–[6]. During the larval stages, cells in wing imaginal discs proliferate exponentially, fostered by nutrients obtained from the hemolymph and gas exchange enhanced by the tracheal system. Larval care thereby permits the expansion of the disc cell population by a factor of thousand over a period of four days [1], [2], [7], [8]. During the initial proliferative phase, cell doubling is completed within about eight hours. Progression through the cell cycle appears to slow down about twofold during the larval stages [9]. In parallel, the epithelial disc pocket that remains connected to the larval body wall via a stalk region differentiates into a squamous sheet, the peripodial membrane, above a pseudostratified columnar sheet, the disc proper [10]. In addition, accompanying disc cell proliferation a series of folds develops in a stereotypic pattern.

At the time the wing imaginal discs are segregated from the prospective larval epidermis during embryogenesis, the epithelial pockets are already separated into an anterior (A) and a posterior (P) compartment [4], [11]. These compartments are specified by the state of *engrailed* (*en*) expression that is clonally propagated. The differential state of *en* expression (off in A, on in P) prevents cell mixing between the compartments. Moreover, interactions between A and P cells result in *decapentaplegic (dpp)* expression in a stripe of A cells along the A/P boundary. The secreted *dpp* gene product functions as a morphogen regulating growth and pattern formation. During the larval stages, an additional compartment boundary develops perpendicular to the A/P boundary, separating a dorsal (D) from a ventral (V) compartment. Cell interactions across the D/V compartment boundary regulate the expression of *wingless* (*wg*) which encodes another secreted morphogen crucial for pattern formation that co-operates with Dpp in growth control.

Beyond morphogens, wing development is also controlled by hormones. Nutrition controls the production and secretion of *Drosophila* insulin-like peptides (Dilps), which stimulate wing imaginal disc cell growth by activation of the insulin receptor signaling pathway [12]–[14]. In addition, the molting hormone 20-hydroxyecdysone is of paramount importance. At the end of the third and final larval instar, a pulse of high levels of 20-hydroxyecdysone production induces metamorphosis. During the pupal stages, cell proliferation in wing imaginal discs comes to a halt and morphogenetic processes remodel the disc epithelium [15], [16]. The central wing pouch region around the intersection of the A/P and D/V boundaries is folded into the double layer epithelium that differentiates into the adult wing blade. Other parts of the wing imaginal discs are transformed into the wing hinge and notal structures.

Apart from the limited structural complexity of the *Drosophila* wing, it is primarily the continuous sophistication of precise genetic methodology and in particular clonal analyses that have sustained the remarkable progress in understanding wing development and developmental biology in general. The great majority of analyses with *Drosophila* wing imaginal discs have relied on observations in fixed samples. Much of important recent progress in cell and developmental biology, however, is based on tracking cell and tissue behavior over time with in vivo imaging. Application of this powerful approach to *Drosophila* wing development is hampered by several difficulties. In vivo imaging of wing imaginal discs in living larvae requires their immobilization. Although immobilization methods have been applied recently [17]–[19], imaging inside the larva cannot provide fully optimal optical resolution. Several studies have therefore applied in vivo imaging with dissected wing imaginal discs in culture [20]–[30]. While these previous publications illustrate the great potential of this culture approach, we demonstrate here most clearly that the applied culture conditions are completely inadequate to maintain imaginal discs in a healthy state, compatible with normal long term growth and development. However, already 50 years ago, it was demonstrated definitively that dissected wing imaginal discs will continue to grow and develop after transplantation into the abdomen of adult females [31]. The search for culture conditions supporting long term development of wing imaginal discs in vitro has also been started early on [32], [33] without widely reproducible success, even though the establishment of stable disc-derived cell lines was accomplished [34]–[36]. In contrast to *Drosophila*, long term culture of wing imaginal discs from lepidopteran species has been more successful. The protocols for cultivation of wing discs from the butterfly *Precis coenia* [37] and tobacco hornworm *Manduca sexta* [38] support disc growth over several days.

Here, we report the results of our work towards establishment of culture protocols allowing long term growth and development of isolated *Drosophila* wing imaginal discs in vitro. For an improved understanding of why current culture conditions might be inadequate, we have characterized cell proliferation and cell cycle progression in cultured discs. We demonstrate that cell proliferation in cultured discs is inhibited rapidly and dramatically, not by a defined arrest at a specific cell cycle checkpoint but rather after severe failure of basic cellular processes like transcriptional activation. With co-culture and transplantation experiments, we exclude that the failure of long term disc growth and development is caused by trivial technical failures. We evaluate the role of various growth promoting pathways and test the suitability of variant media, including hemolymph and co-culture with various larval organs. Although not a complete solution, we report that medium supplementation with high levels of insulin in combination with a rapid and gentle partial dissection of wing imaginal discs is more effective in delaying the cell proliferation arrest in culture than previously described approaches.

## Materials and Methods

### Fly strains

We used *w* mutant strains with insertions of the following previously transcribed transgenes: *P{w{+mC} = PTT-un1}G00044 ( = Lac-YFP)* [39], *P{w{+mC} = UAS-E2F1}5A, P{w{+mC} = UAS-DP}8C* [40], *P{w{+mC} = UAS-puckered 2A}* [41], *P{w{+mC} = UAS-Ras^G12V^}* [42], *P{w{+mC} = UAS-yki.S111A.S168A.S250A.V5} attP2 ( = UAS-Yki^3A^)* [43], *P{w{+mC} = UAS-GFP}*, *P{w{+mC} = UAS-mCherry-NLS}* [44], *P{w{+mW.hs} = en2.4-GAL4}e16E* [45], *P{w{+mC} = tubP-GAL80^TS^}20* [46], *P{GawB}ap^md544^ ( = ap-GAL4)* and *P{GawB}nubbin-AC-62 ( = nubbin-GAL4)* [47], [48]. In addition, we used stocks with the following mutant alleles: *fs(1)K10^1^/Basc* [49]; *Bc^1^/In(2LR)Gla, wg^Gl-1^, Bc^1^* [50], [51]. A pUASt construct was used for the production of the strain with the transgene insertion *P{w{+mC} = UAS-nlsCdt1N101EBFP2-T2A-nlsCycBN96-nlsCycBN285tdTomato-T2A-EGFPPCNA} II.1* ( =  *UAS-RGB* cell cycle tracker). Detailed information concerning this pUASt construct will be provided upon request.

### Culture media and cell lines

We used either Mcl8 [34] or WM1 [30] medium. Mcl8 is composed of Shields and Sang M3 (#S8398, Sigma Aldrich) supplemented with 1 ng/ml 20-hydroxyecdysone, 5 µg/ml bovine Insulin, 5% fly extract, 2% fetal bovine serum (FBS) and 1% Penicillin/Streptomycin (#15140, Gibco). WM1 is composed of Schneider's medium (#21720, Gibco) supplemented with 5% fly extract, 6.2 µg/ml bovine insulin and 1% penicillin/streptomycin (#15140, Gibco). Stock solutions of medium supplements were prepared as follows. 20-Hydroxyexcdysone powder (#H5142, Sigma Aldrich) was dissolved in pure ethanol and stored as stock solution of 5 µg/ml at 4°C. Bovine insulin powder (#I5500, Sigma Aldrich) was dissolved in acidified water (1 µl of 32% HCl in 1 ml water) to arrive at a stock solution (10 mg/ml) which was stored for no longer than one week at 4°C. Chemically synthesized *Drosophila* insulin-like peptide 2 (Dilp2) was kindly provided by H. Stocker (ETH Zurich, Switzerland). Basement Membrane Extract (BME) (#3430-001-02, Cultrex) was thawed over night at 4°C. When used at high concentrations (100%) 50 µl of BME were directly pipetted on a 35 mm culture dish and incubated for 30 min at 37°C. Cultured wing imaginal discs were injected into the resulting gel and covered with additional Mcl8. When used in low concentrations (4%), BME was directly dissolved in Mcl8. Fly extract [34] was kindly provided by S. Restrepo (University of Zurich, Switzerland). Hemolymph was isolated from *Bc/Bc Gla* third instar larvae as described recently [52].

The cell lines Kc [53] and S2R+ cells [54] were cultured at 24°C in Schneider's medium supplemented with 10% heat-inactivated fetal bovine serum, 100 units/ml penicillin and 100 mg/ml streptomycin. A construct (pUbiqPry-nlsCdt1N101EBFP2-T2A-nlsCycBN96-nlsCycBN285tdTomato-T2A-EGFPPCNA-hgr  =  pUbi-*RGB* cell cycle tracker) driving expression of fluorescent proteins under control of the *Ubi-p63E* promoter for tracking cell cycle progression was used for transfection and selection of S2R+ cells with stable insertion. Detailed information concerning pUbi-*RGB* cell cycle tracker will be provided upon request. FuGENE HD reagent was used for transfection. Two days after transfection cells were split and hygromycin (Sigma) was added for selection (300 µg/ml).

### Cultivation of wing imaginal discs

Flies for egg collections were raised on standard food at 25°C. Eggs were collected for two hours followed by ageing for 100 hours at 25°C unless specified otherwise. Larvae were washed out of the fly food and surface sterilized in 70% ethanol. Excess fluid was absorbed on a filter paper and larvae were washed once in sterile PBS (137 mM NaCl, 2.7 mM KCl, 1.47 mM KH2PO4, 6.46 mM Na2HPO4, pH 7.4). After dissection in Mcl8, wing imaginal discs were washed once in fresh medium before transferring them in 30 µl Mcl8 into a 35 mm dish in which the final culture volume was adjusted to 130 µl. Culture dishes were prepared essentially as described [30]. Briefly, a circular opening with a diameter of 14 mm was cut out from the center of the culture dish bottom. The resulting opening was covered with a glass cover slip that was sealed at the periphery to the outside of the bottom of the culture dish with Silicone rubber (Elastosil E41; Wacker Chemie AG). The cover slip was coated with 0.1% (w/v) Poly-L-Lysine (#P8920, Sigma Aldrich) for 30 minutes to avoid floating of discs during coverage with a cell culture insert. Typically, about 10 discs were cultured together at 25°C.

For incubation of cultured discs in hypoxia (5% oxygen) or hyperoxia (60% oxygen), culture dishes were placed in acrylic glass containers with in- and outlets with air-tight seals. At the start of incubations, containers were flushed with appropriate gas mixtures of nitrogen and oxygen (PanGas AG, Dagmarsellen, Switzerland). After incubation, wing imaginal discs were fixed immediately in 4% PFA.

### Transplantation of wing imaginal discs

Early third instar larvae, raised on standard food at 25°C, were surface sterilized in 70% ethanol and washed once in PBS before dissection. Typically, groups of eight to ten wing imaginal discs were dissected and stored on ice before transplantation. We used *fs(1)K10* homozygous females as transplant hosts. Adult hosts were narcotized for few seconds in ether and placed on a double scotch tape with the wings down. A fine glass capillary was filled with PBS and the dissected wing imaginal disc was injected into the female host through fast penetration of the abdomen. Adult hosts were then transferred to fresh food vials stored horizontally. For recovery of transplanted discs, female abdomens were opened in PBS and imaginal discs were removed. Total cell numbers of each disc were analyzed after tissue dissociation in a 30 µl drop of 0.35 M citric acid followed by cell counting in a Thoma chamber [55]. This method was also used for the determination of the number of cells in isolated wing imaginal discs before transplantation. While one of the two discs dissected from each larva was transplanted, the other was used for determination of cell number before transplantation.

### Wounding

Larvae were surface sterilized as described above. After a single wash in PBS, larvae were briefly dried on filter paper, placed on a glass slide and narcotized for about 30 seconds in a bottle containing a cotton ball soaked with ether. Larvae were then transferred onto a new slide and moistened with PBS. Wounding was performed with a fine glass needle of about 50 µm in diameter. The larval cuticle was penetrated at the posterior end. For repeated wounding, the whole procedure of anesthesia, wounding and recovery was repeated three times with intervals of 15 minutes. Wounded larvae were stored on moist filter paper in fresh food vials until dissection of wing imaginal discs.

### EdU labelling

5-ethynyl-2′-deoxyuridine (EdU) reagent (#C10338, Invitrogen) was added to the culture medium to a final concentration of 5 µM. Discs from Lac-YFP larvae (if not stated otherwise) were incubated for 30 minutes before fixation with 4% formaldehyde in PBS (PFA). Fixative was removed and discs were washed once with blocking solution (PBS containing 3% FBS), followed by 20 minutes incubation in PBT (PBS containing 0.5% Triton-X-100). Another washing step was performed with blocking solution before the “click-it” reaction cocktail with Alexa 555 dye was added. Discs were incubated for 30 minutes, protected from light. The reaction cocktail was removed and discs were washed once with blocking solution. Thereafter incubation with primary antibodies was performed.

### Antibody staining

Wing imaginal discs were fixed in 4% PFA for 20 minutes and washed in PBT. After 30 minutes of incubation in blocking solution, incubation with primary antibodies was performed for 90 minutes, followed by three washing steps in PBT (20 minutes each). Secondary antibodies were added and incubated for 60 minutes in blocking solution, again followed by three washing steps in PBT (20 minutes each). DNA was stained with Hoechst 33258 (1 µg/ml in PBS) for 4 minutes. A final washing step was performed with PBS for 20 minutes before wing discs were finally mounted in mounting medium (70% glycerin; 50 mM Tris-HCl pH 8,5; 10 mg/ml propyl gallate; 0,5 mg/ml phenylendiamine).

The following primary antibodies were used: rabbit anti-Ras (31–45) (#553571, Calbiochem) 1∶1000; rabbit anti-phospho-histone H3 (Upstate) 1∶800; rat anti-phospho-histone H3 (Sigma #H9908, clone HTA28) 1∶5000; mouse monoclonal anti-Fasciclin 7G10 (developed by Corey Goodman and obtained from Developmental Studies Hybridoma Bank, created by the NICHD of the NIH and maintained at The University of Iowa, Department of Biology, Iowa City, IA 52242) 1∶50. The following secondary antibodies were used: goat anti-rabbit Alexa568 (Invitrogen) 1∶500; goat anti-rabbit Alexa488 (Invitrogen) 1∶500; goat anti-rabbit Cy5 (Jackson Immuno Research) 1∶500.

### Microscopy and quantitative image analysis

Live imaging with wing imaginal discs was performed on an inverted laser scanning confocal microscope (Olympus Fluoview1000). A 60x oil objective was used for acquisition of z-stacks with 1 µm spacing between focal planes every 330 seconds.

Microscopy of fixed samples was performed on an inverted wide-field fluorescence microscope (Zeiss CellObserver HS). Images of whole wing imaginal discs were acquired with a 20x objective. Image stacks of the wing pouch region were acquired with a 40x objective with 1 µm spacing between focal planes. For quantification of EdU incorporation, average projections from five sections were generated using ImageJ (http://rsbweb.nih.gov/ij/) (n≥14 imaginal discs). A region of interest of constant size covering the majority of the central pouch region was selected at a comparable location in all of the projections, followed by determination of mean pixel intensity. For visual inspection of image stacks we also used Imaris (Bitplane scientific software). Adobe Photoshop and Adobe Illustrator were utilized for assembly of figures.

To count the number of mitotic cells, we used either of two procedures. A first approach involved the analysis of wing imaginal discs expressing Lac-YFP, which is localized to the septate junctions. In live as well as in fixed preparations of such discs, mitotic cells can be identified because of their characteristic rounded shape readily revealed by the YFP signals. Alternatively, fixed imaginal discs were immunolabeled with antibodies against phospho-histone H3. Image stacks were acquired with a 40x/0.75 objective and a Zeiss CellObserver HS microscope. A first focal plane in these stacks was acquired at the plane of the peripodial membrane. Additional z sections with 1 µm spacing were acquired in direction towards the basal side of the disc epithelium. By visual analysis of the image stack, all mitotic cells within the central pouch region delimited by the nearest circumferential fold were counted. Moreover, as an approximate estimate for the evaluated area of disc epithelium, the area delimited by the circumferential folds was measured in a maximal projection of the image stack. The average area of this region was found to be 1.79×10^4^ µm^2^ (s.d. = 2670; n = 21) in wing imaginal discs isolated from larvae 100 hours after egg deposition (AED). In discs isolated at 104 and 118 hours AED, the average area was found to be 2.38×10^4^ µm^2^ (s.d. = 4192; n = 10) and 3.17×10^4^ µm^2^ (s.d. = 4787; n = 9), respectively. To facilitate comparisons between different time points, the observed total number of mitotic cells within the central pouch region delimited by the nearest circumferential fold was divided by the size of the analyzed area in a given disc and multiplied by the reference area for which we used the average size of the analyzed area at 100 hours AED (1.8×10^4^ µm^2^). Therefore, the resulting numbers describe a mitotic index and not the total number of mitotic cells.

EGFP expression levels in wing imaginal discs from *P{w{+mW.hs} = en2.4-GAL4}e16E*, *P{w{+mC} = tubP-GAL80^TS^}20*, *UAS-EGFP* larvae after temperature shift from 23 to 29°C were measured in the central pouch region from average projections of four sections (z-step size: 2 µm; n≥6 discs per time point). Average signal intensity obtained in the anterior compartment was subtracted as background from the average signal intensity observed in the posterior compartment.

For statistical analyses, Excel (Microsoft) and OriginPro8.6 (OriginLab) were used. Statistical significance was estimated by t test and is indicated in some figures by one, two or three stars corresponding to p*<0.05, p**<0.01, and p***<0.001, respectively. NS  =  non-significant. The images for documentation of the effects of overexpression of E2f1/Dp, Ras85D^V12^ or Yki^3A^ show representative examples of more than 10 analyzed imaginal discs.

### Immunoblotting

Kc cells were solubilized in SDS-PAGE sample buffer. Total cell extracts were resolved by SDS-PAGE and analyzed by immunoblotting using the following antibodies: rabbit polyclonal anti-Phospho-*Drosophila* Akt (Ser505) (#4054S, Cell Signaling Technology) 1∶1000; mouse monoclonal anti-alpha-Tubulin DM1A (#T9026, Sigma Aldrich) 1∶50,000; goat anti-mouse POD (Jackson Immuno Research) 1∶1000; goat anti rabbit HRP (Jackson Immuno Research) 1∶10,000.

## Results

### In vitro culture of wing imaginal discs results in a rapid decrease in the number of mitotic cells

**Figure 1:**
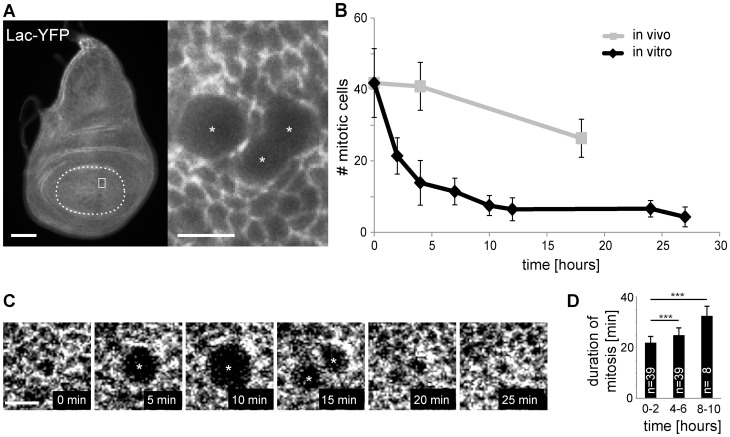
Frequency and duration of mitosis in cultivated wing imaginal discs. (A) Numbers of mitotic cells were determined in wing imaginal discs expressing *Lac-YFP*. The central pouch region delimited by the nearest circumferential fold (dashed line in left panel) was analyzed. Mitotic cells (asterisks) were identified based on their characteristic rounded shape as illustrated by the region (white square in left panel) shown at higher magnification (right panel). Scale bars correspond to 50 µm (left) and 5 µm (right), respectively. (B) The average numbers of mitotic cells in discs fixed immediately after dissection at either 100, 104, or 118 hours AED (in vivo) were compared to the average numbers observed in discs cultured after dissection at 100 hours AED for the indicated time (in vitro). Numbers of mitotic cells were normalized to the reference region (average size of the central pouch at 100 hours AED). n≥8 discs per time point; s.d. indicated. t = 0 in the graph corresponds to 100 hours AED. (C, D) Time lapse imaging as illustrated (C; scale bar = 5 µm) was used to determine the duration of mitosis in discs during the first two hours in culture (0–2 hours) as well as during later times. Bars indicate average duration with s.d., *** p<0.001 (t test).

To evaluate the effects of in vitro culture on cell cycle progression in wing imaginal discs, these were dissected from larvae and incubated in Mcl8, a medium that has been used most frequently for imaginal disc culture so far. The number of mitotic cells detected during cultivation in vitro were counted and compared with those observed in vivo (Fig. 1). For the identification of mitotic cells, we exploited the fact that cells entering mitosis round up on the apical side of the disc epithelium. Mitotic cells can therefore be identified readily in discs expressing Lachesin (Lac)-YFP which localizes to the apico-lateral septate junctions [39], [56] (Fig 1A). In discs dissected 100 hours after egg deposition (AED), we counted on average 42 mitotic cells (+/−9.6 s.d., n = 17) within the central wing pouch region delimited by the nearest circumferential fold (Fig. 1A, B). During culture of discs in vitro, the number of mitotic cells decreased very rapidly and dramatically (Fig. 1B). In contrast, during development in vivo the decrease was far more limited, as revealed by counts obtained from discs fixed immediately after dissection at 104 and 118 hours AED, respectively (Fig. 1B). The observed limited decrease of mitotic cells in vivo as well as the drastic decrease in vitro is consistent with earlier reports [7], [30], [57], [58].

By time lapse imaging, we evaluated whether the duration of mitosis was also affected by disc culture (Fig. 1C, D). Mitoses observed after 4–6 hours of cultivation were slightly but significantly slower than those observed within the first two hours of cultivation (Fig. 1D). Moreover, the few mitoses after longer cultivation (8–10 hours) appeared to be slowed down further (Fig. 1D) but in every case they were apparently completed.

### In vitro culture of wing imaginal discs results in a rapid inhibition of S phase

**Figure 2:**
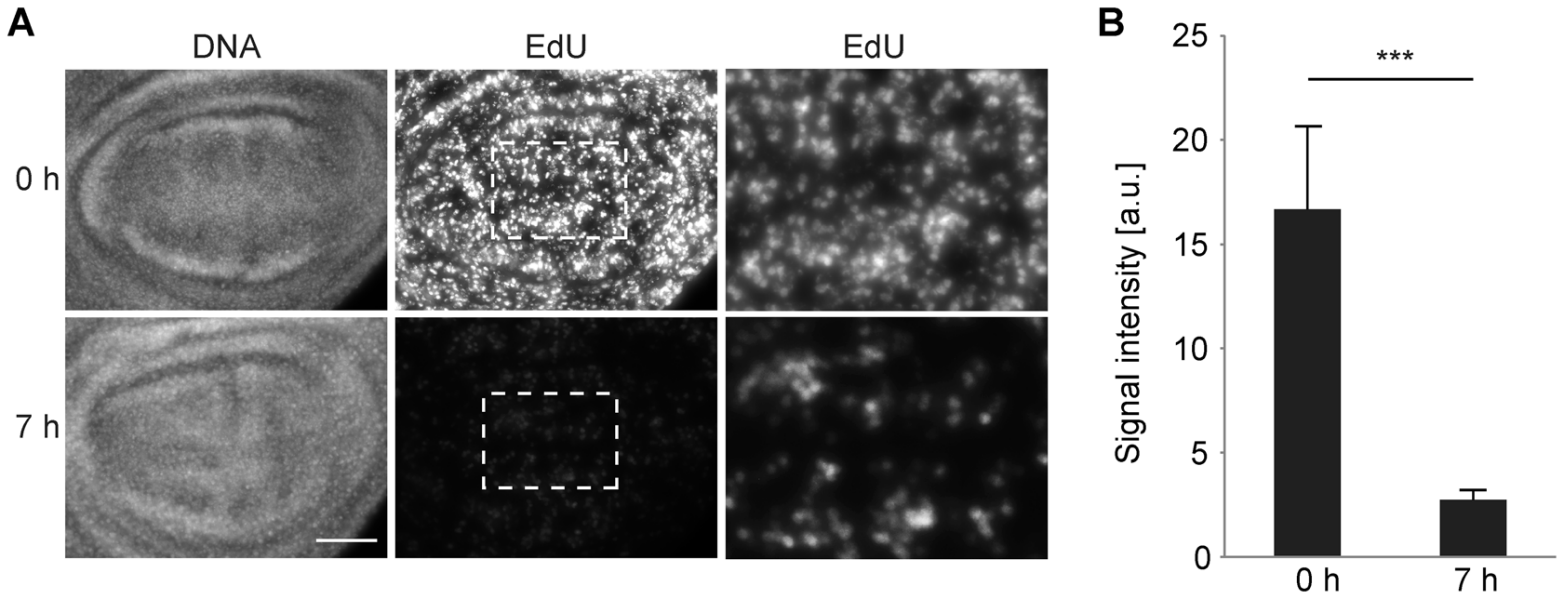
Progression through S phase in cultured wing imaginal discs. (A) Wing imaginal discs dissected 100 hours AED were pulse labeled with EdU (30 minutes) either immediately (0 h) or after 7 hours of cultivation (7 h). After fixation and EdU visualization, discs were double labeled with Hoechst (DNA). EdU signals in wing pouch regions after identical acquisition and processing are shown in the second row. Central regions (white frames in the second row) are shown at higher magnification after contrast maximization in the third row. Scale bar = 40 µm. (B) EdU signal intensities integrated over the pouch region were quantified. Bars indicate average integrated intensity and whiskers s.d.; n≥14 discs; *** p<0.001 (t test).

In principle, the rapid and dramatic decrease in the number of mitotic cells might result from an inhibitory effect of cultivation on exclusively the G2/M transition. Alternatively, culture conditions might rapidly disrupt also other events during interphase. To resolve this issue, we performed EdU pulse labelling [59]. A decrease in the number of EdU labelled cells but not in the intensity of EdU signals in labelled cell nuclei is expected, if culture conditions are incompatible with progression through either G1 or G2 without affecting S phase progression. In contrast, reduced nuclear signal intensities are expected, if culture conditions slow down progression through S phase. Indeed, compared to discs that were EdU pulse labelled immediately after dissection, nuclear signals were found to be strongly reduced after identical labelling following 7 hours of cultivation (Fig. 2). We conclude that culture conditions rapidly impair progression through S phase. Moreover, the fact that the observed drop in the number of mitotic cells during culture was so rapid (Fig. 1B) indicated that events after completion of S phase are affected as well. If only progression through S but not G2 was slowed down by culture conditions, cells already in G2 at the start of disc culture would still divide on time, resulting in a lag phase of a few hours corresponding to the reported duration of G2 in wing imaginal discs at around 100 hours AED [60] before an eventual drop in the number of mitotic cells.

### A novel cell cycle reporter confirms incompatibility of culture conditions with cell cycle progression in wing imaginal discs

**Figure 3:**
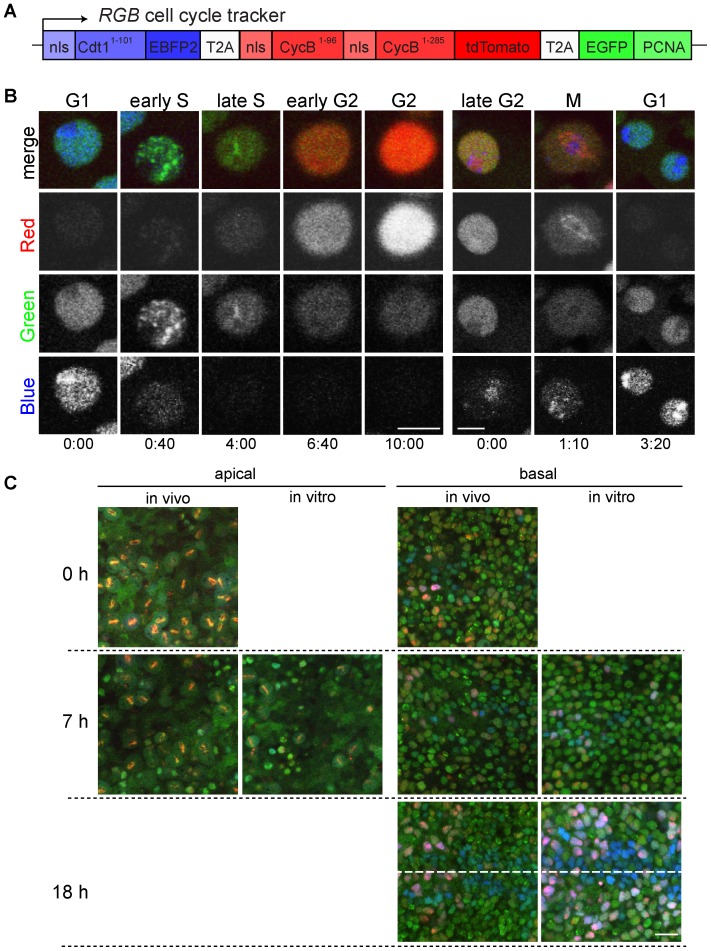
A novel *RGB* cell cycle tracker reveals inhibition of cell cycle progression in cultured discs. (A) The transcript expressed from the *RGB* cell cycle tracker transgene generates three separate proteins because of the intervening T2A cis-acting hydrolase elements. The three proteins are fused to different fluorescent proteins, EBFP2 (blue), tdTomato (red) and EGFP (green). Their intracellular localization is dictated by nuclear localization signals (nls). Moreover, the PCNA part enforces localization in a subnuclear pattern during S phase. Degradation signals result in proteolysis during S phase (Cdt1^1–101^) or late M and G2 (CycB^1–96^ and CycB^1–285^), respectively. (B) Time lapse in vivo imaging of peripodial cells in cultured *en-GAL4 UAS-RGB* wing imaginal discs. In the left series of still frames, a cell progressing from G1 through S into G2 is displayed. In the right series of still frames, a cell progressing from G2 through M into G1 is shown. Time is given in hours∶minutes. Scale bars  = 5 µm. (C) The effect of culture on cell cycle progression was analyzed with *nub-GAL4 UAS-RGB* wing imaginal discs. Discs fixed immediately after dissection at 100, 107 or 118 hours AED are labeled "in vivo", while those dissected at 100 hours AED followed by cultivation for 7 or 18 hours before fixation are labeled "in vitro". Representative images of the central pouch region at either an apical focal plane containing mitotic cells (left side) or a basal focal plane with interphase cells in the disc proper (right side) are shown. The dashed line indicates the future wing margin. Scale bar  = 10 µm.

To verify the inhibitory effects of culture conditions on progression through the cell cycle, we analysed wing imaginal discs expressing a novel cell cycle reporter transgene. This *RGB* cell cycle tracker transgene (Fig. 3A) results in expression of a single mRNA that generates three distinct fusion proteins because of separating T2A cis-acting hydrolase elements within the encoded open reading frame [61]. The first fusion protein, nlsCdt1^1–101^-EBFP2 is a blue fluorescent nuclear protein that is degraded specifically during S phase because it contains the N-terminal region of Cdt1 which includes a Cul4Cdt2-dependent degron [62]. The second fusion protein, nlsCycB^1–96^-nlsCycB^1–285^-tdTomato, is a red nuclear protein that is degraded specifically during late M and G1 because it includes Cyclin B regions containing APC/C-dependent D box degrons [63], [64]. The third fusion protein, EGFP-PCNA, is a green nuclear protein with a characteristic distinct subnuclear pattern during S phase reflecting association with replication factories [65].

Validation of this *RGB* cell cycle tracker was performed with a construct under control of a constitutive promoter (*Ubi-p63E*) in stably transfected S2R+ cells (Fig. S1). These cells proliferate readily in culture in contrast to wing imaginal discs and are therefore readily amenable to long term time lapse imaging. As expected, after completion of mitosis, cell nuclei displayed homogenous blue and green signals during G1, until the blue signals disappeared rapidly, concomitant with redistribution of the green signal into a distinct granular pattern at onset of S phase. Red signals started to accumulate in late S when EGFP-PCNA was concentrated into few large intranuclear clusters. Red signal intensities increased during G2 when EGFP-PCNA was again homogenously distributed throughout the nucleus. Late in G2 also blue signals were observed to re-accumulate again. During early mitosis, the red protein was enriched on spindles before its degradation during exit from mitosis, when the blue and green proteins re-accumulated in the nuclei after their homogenous distribution throughout the cells during mitosis.

The distinctive colour combinations and subnuclear patterns detected during cell cycle progression in the *RGB*-S2R+ cell line were also observed after expression of an *UAS-RGB* transgene in wing imaginal discs. They could be detected particularly well in the flat cells within the peripodial membrane (Fig. 3B). While we could not track peripodial cells through complete cell cycles in disc cultures, we could readily image for periods close to a complete cycle and detected the same rapid and characteristic changes indicative of the different cell cycle transitions described above.

To study effects of culture conditions on cell cycle progression in disc epithelial cells, we expressed *UAS-RGB* using *nubbin-GAL4* and fixed imaginal wing discs either immediately after dissection or after additional cultivation for 7 or 18 hours in vitro (Fig. 3C). Analysis of the apical regions of the central pouch disc epithelium, which contain the mitotic cells, fully confirmed that in vitro cultivation results in a rapid decrease in the number of mitotic cells, already apparent after 7 hours of cultivation. Within more basal regions, which contain the interphase cells, the colour pattern was not yet strikingly different at the 7 hour time point, but after 18 hours of cultivation we observed a strong increase in the number of cells that had strong red and blue signals (Fig. 3C), indicating a delay or arrest in G2. In addition, a stripe of cells with strong blue but not red signals was evident at this late time point along the future wing margin in particular in the anterior compartment. This zone of non-proliferating cells (ZNC) is known to accumulate cells arrested in G1 in late larval wing imaginal discs during normal development [13].

### Overexpression of E2f1/Dp does not restore cell cycle progression in cultured wing imaginal discs

**Figure 4:**
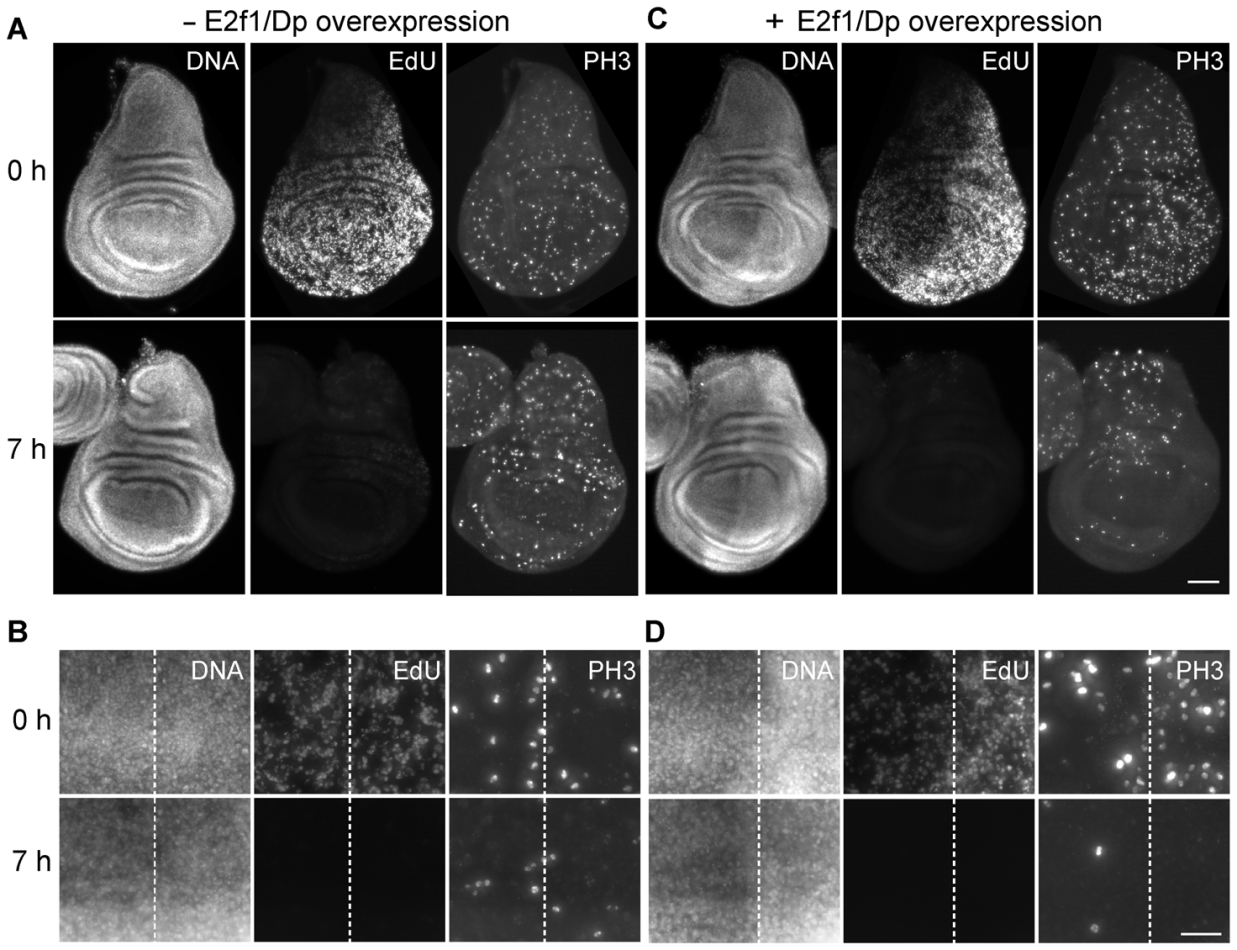
Effects of E2f1/Dp overexpression on cell cycle progression during wing imaginal disc culture. Imaginal discs were dissected from larvae with single copies of the transgenes *en-GAL4*, *UAS-E2f1*, *UAS-Dp*, and *tub-GAL80^ts^*. These larvae were either grown constantly at 23°C (A, B; - E2f1/Dp overexpression) or at 29°C for the final 18 hours before disc dissection (C, D; + E2f1/Dp overexpression). Dissected wing imaginal discs were labeled with EdU (EdU), anti-PH3 (PH3) and a DNA stain (DNA) either immediately (0 h) or after 7 hours of cultivation at 25°C (7 h). Complete imaginal discs (A, C; scale bar  = 50 µm) and high magnification views of the central pouch region (B, D; scale bar  = 20 µm) are shown with dashed lines indicating the boundary between anterior and posterior compartment, in which *en-GAL4* driven *UAS* transgene expression occurs at 29°C.

E2f1/Dp heterodimers are known to regulate the transcription of key cell cycle regulators like Cyclin E and String/Cdc25 phosphatase, as well as many other genes required for progression through the cell cycle [40], [66], [67]. The reported acceleration of progression through the cell division cycle resulting from overexpression of E2f1/Dp has clearly demonstrated the crucial role of this transcription factor in wing imaginal discs [40]. Therefore, we evaluated whether E2f1/Dp overexpression might suppress the inhibitory effects of culture conditions on cell cycle progression in cultivated wing imaginal discs. For overexpression we used *UAS-E2f1* and *UAS-Dp* transgenes in combination with *en-GAL4*. Thereby overexpression is achieved in the posterior compartment of the wing imaginal disc but not in the anterior compartment which therefore can be used as an internal control. Moreover, for temporal control of overexpression we used *tub-GAL80^ts^*. Therefore, when larvae were grown at a low temperature (23°C), E2f1/Dp overexpression was insignificant as suggested by EdU pulse labeling immediately after disc dissection in combination with double labeling with anti-phospho-histone H3 (PH3), a marker of mitotic cells. Signal intensities and number of labeled cells were comparable in the anterior and posterior compartment (Fig. 4A, B, 0 h). In contrast, when larvae were shifted from 23 to 29°C during the final 18 hours before disc dissection, we observed increased labeling with EdU and anti-PH3 in the posterior compartment (Fig. 4C, D, 0 h), as expected [40]. Importantly, after 7 hours of disc cultivation (Fig. 4A-D, 7 h), EdU incorporation and anti-PH3 staining were strongly reduced to the same extent throughout the disc, also after E2f1/Dp overexpression had been driven up in the posterior compartment before the onset of disc culture (Fig. 4C, D, 7 h). These results indicate that E2f1/Dp overexpression cannot by-pass the cell cycle block induced by imaginal disc cultivation.

### In vitro culture of wing imaginal discs results in a rapid inhibition of GFP transgene inducibility

**Figure 5:**
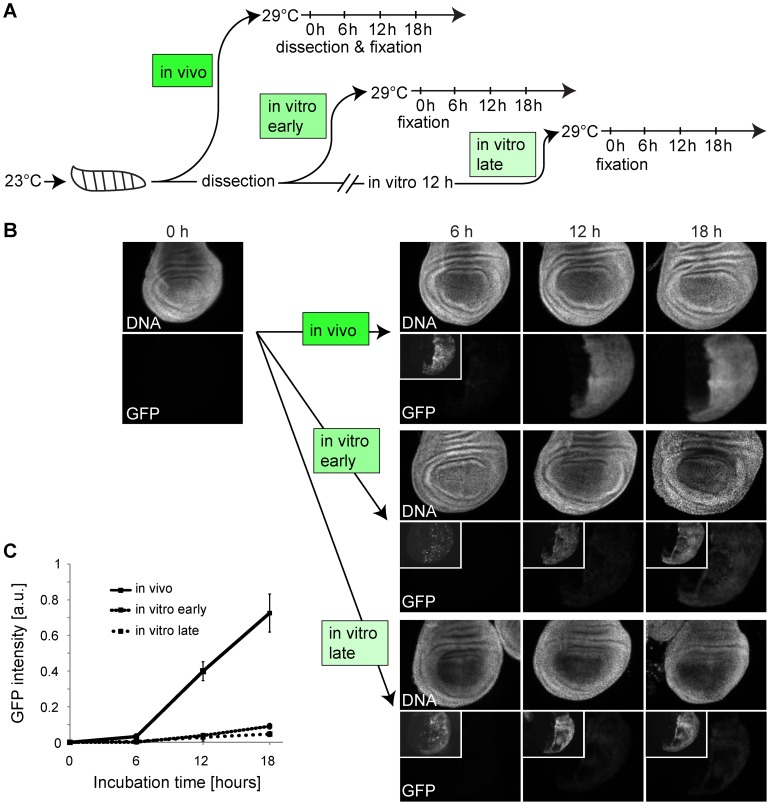
Effects of culture condition on induction of *UAS-EGFP* expression in wing imaginal discs. (A) After development at 23°C, an aliquot of *en-GAL4 UAS-EGFP tub-GAL80^ts^* larvae were shifted to 29°C for the indicated times before dissection and microscopic analysis of wing imaginal discs (in vivo). Another aliquot of these larvae was used for dissection of wing imaginal discs that were either immediately shifted to 29°C (in vitro early) or after 12 hours of cultivation at 23°C (in vitro late) before fixation at the indicated times. (B) Wing pouch region of imaginal discs from *en-GAL4 UAS-EGFP tub-GAL80^ts^* larvae after induction of *UAS-EGFP* expression by shifts from 23°C to 29°C as described in (A). Insets display higher contrast to reveal weak signals. Scale bar  = 50 µm. (C) Quantification of EGFP signals in the posterior compartment of the pouch region of imaginal discs from *en-GAL4 UAS-EGFP tub-GAL80^ts^* larvae after induction of *UAS-EGFP* expression by shifts from 23°C to 29°C as described in (A) and illustrated in (B).

As culture conditions rapidly inhibited progression through the cell cycle in a way that could not be suppressed by E2f1/Dp overexpression, it appeared likely that cellular functions in cultured wing imaginal discs are compromised in a rather pleiotropic manner. To evaluate whether gene expression might also be among the affected processes, we analyzed the expression of *UAS-EGFP* induced by *en-GAL4* after inactivation of *tub-GAL80^ts^* by a shift from 23 to 29°C (Fig. 5A). When *UAS-EGFP* expression was induced in control experiments by shifting intact larvae to 29°C, EGFP signal intensities were observed to increase strongly in wing imaginal dics dissected and fixed at various times after the shift (Fig. 5B, C). In contrast, when discs where dissected first and thereafter shifted to 29°C in culture, we observed a far weaker induction of EGFP expression (Fig. 5B, C). An even weaker induction of EGFP expression resulted when discs were cultured for 12 hours at 23°C before they were shifted to 29°C (Fig. 5B, C). We conclude that culture conditions rapidly result in a state that is no longer compatible with efficient GAL4-mediated induction of *UAS-EGFP* expression.

### The procedure for isolation of wing imaginal discs does not cause irreversible damage

The results of our analysis of gene inducibility in combination with our findings concerning cell cycle progression suggested that cultivation of imaginal discs provokes considerable stress. Various stressors including wounding are known to activate the Jun NH2-terminal kinase (JNK) signal transduction pathway [68]–[70]. JNK signaling has context-dependent consequences. During imaginal disc regeneration, it promotes blastema formation and proliferation [71]. In contrast, JNK signaling induces apoptosis during correction of developmental errors in wing imaginal discs [72]. Although anti-cleaved Caspase 3 stainings failed to reveal rapid induction of apoptosis within the first 18 hours of in vitro cultivation, it remained conceivable that disc culture might result in JNK pathway activation and thereby interfere with further development of wing imaginal discs in vitro. To evaluate this notion we inhibited JNK signaling by overexpression of *puckered* (*puc*). Puc functions as a JNK phosphatase and potent negative feedback regulator of the JNK pathway [41]. *UAS-puc* was directed exclusively to the posterior compartment using en-GAL4. While *UAS-puc* expression appeared to have minor and apparently non cell-autonomous effects (Fig. S2), it was clearly unable to maintain cell proliferation in cultured wing imaginal discs at a rate comparable to that observed in vivo.

**Figure 6:**
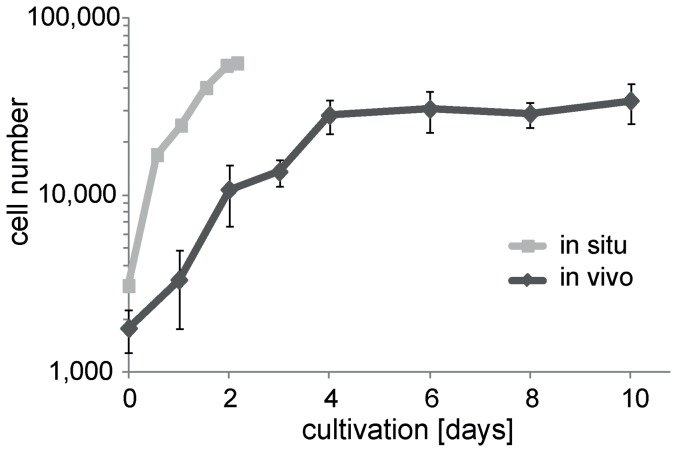
Cell proliferation in wing imaginal discs after transplantation in female abdomina. Wing imaginal discs were isolated from early L3 larvae, but instead of in vitro culture they were immediately transplanted into the abdomen of adult female host flies. Transplanted discs were re-isolated at the indicated times of cultivation in the host and cell counts were determined; cell counts were also determined just before transplantation. Black lines and diamonds (average cell number +/− s.d.; n≥11 per time point) illustrate cell proliferation after transplantation. For comparison cell counts were also determined immediately after isolation of discs from larvae aged for different times (73, 86.5, 98, 110, 120, 125 hours AED). Grey lines and squares (average cell number, n≥8 per time point, s.d. completely covered by the squares) illustrate cell proliferation in situ. The difference in cell numbers at the start of the curves presumably reflects limitations in staging precision.

All our results described so far emphasized that isolated wing imaginal discs are strikingly incapable of developing in vitro. Therefore we considered the possibility that our disc isolation procedure might generate irreversible damage. To evaluate this notion, we cultured our isolated discs under conditions that have previously been proven to be compatible with growth and development of isolated discs in principle. Such permissive conditions can be obtained by transplantation of the isolated imaginal disc into an abdomen of an adult female [7]. To facilitate dissection and re-isolation of discs after transplantation, we used GFP expressing discs (*ap-GAL4, UAS-EGFP*) in combination with host flies that did not express any EGFP. One of the two discs isolated from each donor larva was transplanted, while the other sibling disc was used to obtain a disc cell count. The transplanted discs were re-isolated after defined periods of cultivation in the host abdomen and then also used for disc cell counts. Moreover, for comparison of cell proliferation in transplanted discs with that occurring in situ, we determined disc cell counts after disc isolation at different times AED. These experiments clearly established that the wing imaginal discs generated by our isolation procedure were not irreversibly damaged. After transplantation of these discs, cell proliferation was observed at a rate which was only slightly below that in discs growing unperturbed in situ (Fig. 6). The cell population of transplanted discs underwent a total of four population doublings within 4 days before entering a stationary phase. Cell cycle time during growth in host abdomina appeared to be increased about twofold compared to development in situ (Fig. 6) [9], [40], [60].

While our disc transplantations clearly established that our disc isolation procedure did not irreversibly damage wing imaginal discs, these experiments did not exclude the possibility that transient damage and inhibition of cell proliferation might arise from isolation. The occurrence of a transient inhibition was actually suggested by Bosch and colleagues [71] based on anti-PH3 stainings and BrdU pulse labeling early after disc transplantation. To address potential damage associated with disc isolation, we explored whether wounding of larvae might already be sufficient to cause a rapid inhibition of cell proliferation in their wing imaginal discs. These analyses did not reveal a rapid inhibitory effect of larval wounding on the number of mitotic cells (Fig. S3). We conclude that larval wounding, which evidently cannot be avoided when wing imaginal discs are to be isolated, does not cause damage that necessarily precludes further disc development in culture.

### Evaluation of potential limitations in imaginal disc culture conditions

As our isolation procedure did not irreversibly damage wing imaginal discs, the observed failure of cell proliferation during disc culture might reflect medium deterioration resulting from rapid depletion of essential factors or accumulation of toxic products. To evaluate these possibilities, we co-cultured S2R+ cells with wing imaginal discs in the same culture set up for two days. These co-cultured S2R+ cells were able to proliferate normally (Fig. S4), indicating that the conditions in our disc culture set up are perfectly adequate at least for S2R+ cells. Moreover, although not quantified in detail, co-culture of S2R+ cells did not appear to have beneficial effects on normal long term growth and development of wing imaginal discs in culture.

In principle, the partial pressure of oxygen during in vitro culture of imaginal discs might be a critical determinant. In contrast to S2R+ cells, wing imaginal discs might not be adapted to growth in atmospheric concentrations of oxygen. With mammalian cells in culture, effects of oxygen levels have been demonstrated clearly [73]–[75]. The partial pressure of oxygen to which wing imaginal discs are exposed to in situ is not known. The evident close apposition of trachea with wing imaginal discs might lead to higher oxygen levels in situ than in our culture set up, but as the functionality of some of the disc-associated tracheae is uncertain, the opposite is not excluded either [76]. To evaluate the importance of oxygen levels, we cultured wing imaginal discs in either hypoxic (5% O_2_), normoxic (21% O_2_) or hyperoxic conditions (60% O_2_). Neither hypoxia nor hyperoxia prevented the rapid cell proliferation arrest in culture (Fig. S5).

**Figure 7:**
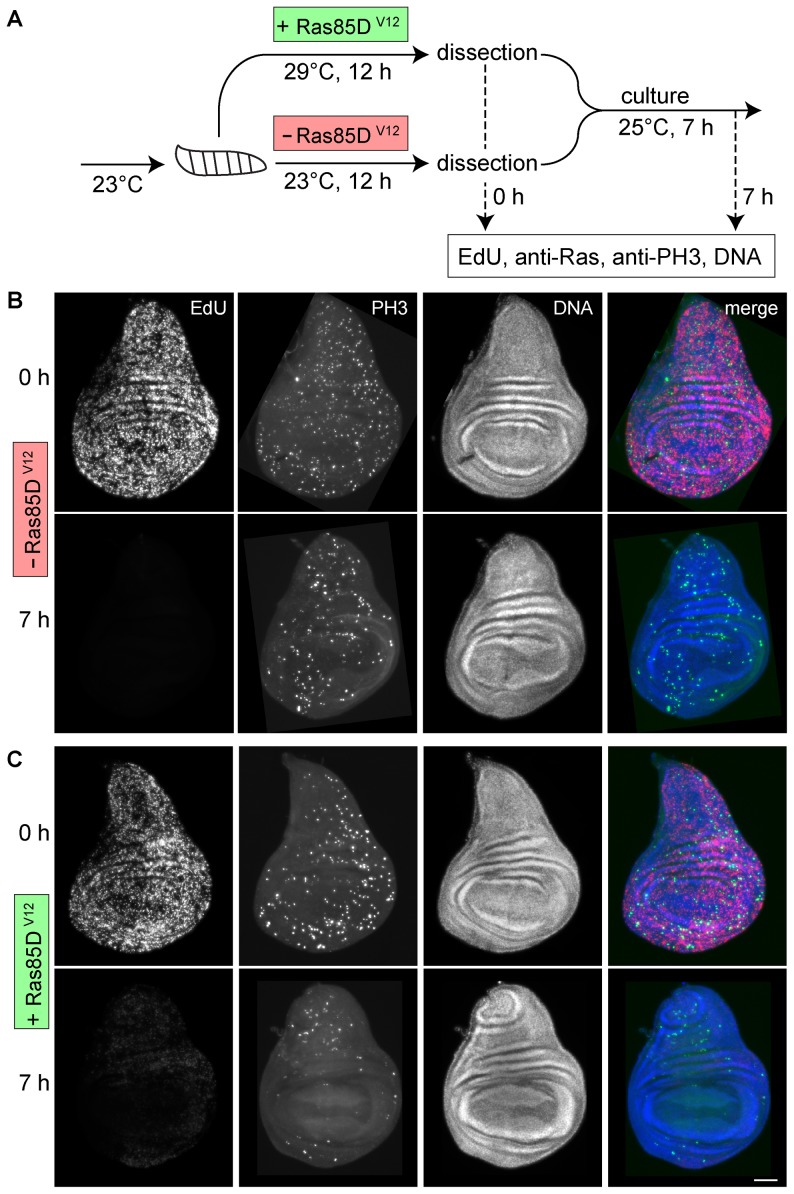
Effects of Ras85D^V12^ expression on cell cycle progression during wing imaginal disc cultivation. (A) *en-GAL4 tub-GAL80^ts^ UAS-Ras85D^V12^* larvae were used for spatially and temporally controlled hyperactivation of the Ras signaling pathway as illustrated schematically. (B, C) Wing imaginal discs from larvae without (B; -Ras85D^V12^) or with (C; +Ras85D^V12^) preceding *UAS-Ras85D^V12^* expression in the posterior compartment were isolated and fixed either immediately (0 h) or after seven hours of cultivation in vitro (7 h) before EdU labelling and staining with anti-PH3 and a DNA dye. Scale bar  = 50 µm.

Constitutive activation or bypass of growth promoting signal transduction pathways by somatic mutations or epigenetic alterations is thought to explain immortalization during derivation of cell lines and thus perhaps also our finding that S2R+ cells but not wing imaginal discs cells proliferate well in our culture set up. Therefore we analyzed whether hyperactivation of two important pathways in wing imaginal discs, Ras and Yorkie, might suppress the cell cycle arrest that is induced by cultivation. Activation of Ras signaling by various growth factors has been demonstrated in many experimental systems. In *Drosophila*, Ras85D is known to regulate cell growth in wing imaginal discs. Cells lacking Ras85D function grow slowly, while growth is enhanced in cells expressing the activated Ras85D^V12^ variant [77]–[79]. Moreover, activated Ras85D^V12^ can also suppress JNK-mediated apoptosis induced by developmental abnormalities like loss of apico-basal cell polarity and wounding [80]–[83] and facilitates the immortalization of primary embryonic cells [84]. Therefore we tested whether Ras85D^V12^ expression prevented the rapid arrest of progression through the cell cycle upon wing imaginal disc culture (Fig. 7A). We used *en-GAL4* in combination with *tub-GAL80^ts^* to activate Ras85D^V12^ expression specifically in posterior compartments after a shift of mid third instar larvae from 23 to 29°C 12 hours before isolation of imaginal discs. In control larvae that were not shifted to 29°C before disc isolation, we were unable to detect increased anti-Ras signals in the posterior compartment (Fig. S6). In contrast, anti-Ras signals were clearly increased in the posterior compartment of discs from larvae that had been shifted to 29°C, already at the start and also after seven hours of disc cultivation (Fig. S6). EdU and PH3 labeling confirmed that Ras85D^V12^ expression in the posterior disc compartment after a temperature shift to 29°C resulted in the expected consequences [78], [79]. In posterior compartment, the number of EdU- and PH3-positive cells was clearly higher than in the anterior compartment at the start of disc cultivation but only in discs from larvae that had been shifted to 29°C (Fig. 7B, C, 0 h). After seven hours of cultivation, EdU incorporation and the number of PH3-positive cells was strongly reduced to a comparable level in both the anterior and the posterior compartment of discs from larvae without and also with preceding shift to 29°C (Fig. 7B, C, 7 h). We conclude that Ras pathway activation is not sufficient to overcome the cell cycle arrest induced by wing imaginal disc cultivation.

Apart from Ras, the Yorkie (Yki) pathway is also crucial for the control of wing imaginal disc growth [85]. Yki functions as transcriptional regulator that is regulated by the Hippo tumor suppressor network. Phosphorylation of Yki is known to inhibit its function as growth promoter and inhibitor of apoptosis. In contrast, expression of a mutant form of Yki (YkiS111A, S168A, S250A  =  Yki^3A^) which can no longer be inhibited by phosphorylation results in imaginal disc overgrowth [43]. Therefore, we tested whether expression of this hyperactive form of Yki is sufficient to prevent the rapid cell cycle arrest that is observed during wing imaginal disc cultivation. We applied the same experimental strategy as described above in case of Ras85D^V12^. Our results suggested that Yorkie pathway activation is not sufficient to overcome the cell cycle arrest induced by wing imaginal disc cultivation (Fig. S7).

### Evaluation of alternative media for culture of wing imaginal discs

**Figure 8:**
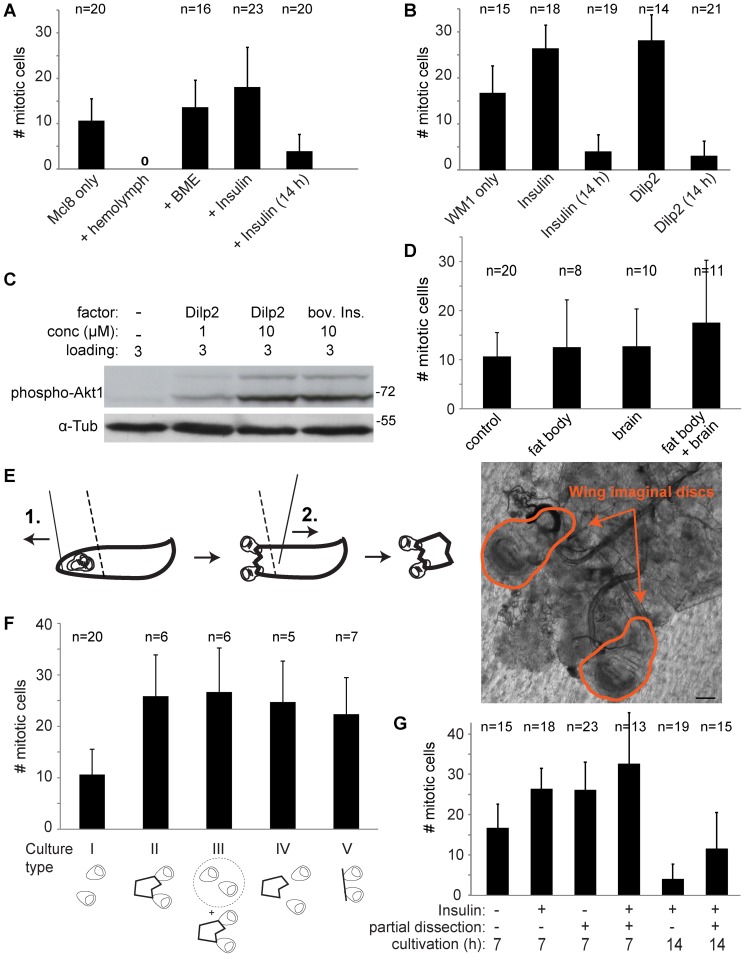
Comparative analysis of different wing imaginal disc culture conditions. (A, B) Effects of medium supplementation with candidate growth promoting factors. Wing imaginal discs were isolated (100 hours AED) and cultured in Mcl8 (A) or WM1 (B) culture media with the indicated supplements: none (Mcl8 only), 25% larval hemolymph (+ hemolymph; n  = 12), 4% BME (+ BME), 35 µM ( = 0.2 mg/ml) bovine insulin (+ Insulin), none (WM1 only), 35 µM bovine insulin (+ Insulin), 0.5 µM Dilp2 (+ Dilp2). After cultivation for 7 hours or in some cases for 14 hours as indicated (14 h), discs were fixed and stained with anti-PH3 for determination of the number of mitotic cells. Bars indicate average number of mitotic cells +/− s.d. (C) To confirm the activity of chemically synthesized Dilp2, Kc cells were treated for 30 minutes as indicated before analysis of total cell extracts by immunoblotting with an antibody against active Akt1 (phospho-Akt1) and with anti-Tubulin (α-Tub) to control loading.(D) Effect of co-cultivation of larval tissues. Wing imaginal discs isolated from larvae 100 hours AED were cultured for 7 hours in Mcl8 without (control) or with additional larval tissues from 10 larvae (fat body, brain, as indicated). The number of mitotic cells was determined after anti-PH3 staining. Bars indicate average +/− s.d.; the number of analyzed discs (n) is indicated. (E, F) Effect of partial dissection of wing imaginal discs. (E) To reduce mechanical stress during isolation of wing imaginal discs and retain larval tissues that might produce endocrine signals, partial disc dissection was performed as schematically illustrated. With a first cut (1., solid line) the very anterior most region of larvae was removed resulting in extrusion of wing imaginal discs, while the larvae was fixed with a forceps (dashed line). With a second cut (2., solid line) the posterior two thirds of larvae were removed. Bright field image of a pair of partially dissected disc is displayed on the right. Scale bar  = 100 µm. (F) Cultures with wing imaginal discs from larvae 100 hours AED were set up in different ways (culture type I-V). In type I, completely dissected discs were cultured for control. In type II, partially dissected discs were analyzed. In type III, completely dissected discs were analyzed after co-culture with partially dissected discs. In type IV, partially dissected discs were separated from the other larval parts before culture. In type V, discs with intact stalks and connections to the tracheal trunk were cultured without any additional larval tissues. The number of mitotic figures present after 7 hours of cultivation in Mcl8 was determined after fixation and anti-PH3 staining. Bars indicate average number of mitotic cells +/− s.d.; the number of discs (n) is indicated. (G) Effect of partial dissection in combination with high insulin. Wing imaginal discs isolated from larvae 100 hours AED either by complete or partial dissection were cultured for 7 or 14 hours in WM1 with or without 0.2 mg/ml bovine insulin as indicated. The number of mitotic cells was determined after anti-PH3 staining. Bars indicate average +/− s.d.; the number of analyzed discs (n) is indicated.

As we were unable to implement long term wing imaginal disc growth in culture by circumventing a potential absence of essential growth factors from the medium by transgenic activation of Ras or Yki pathways, we considered alternative avenues towards a more appropriate disc culture medium. As imaginal discs in situ are exposed to larval hemolymph, this hemocoel fluid appeared to be an obvious choice. Notably, as also confirmed above, isolated discs develop successfully after transplantation into the abdomen of adult females where transplanted discs are also immersed in hemolymph. However, hemolymph cannot be isolated readily in large quantities. Moreover, mass isolation of hemolymph is inextricably linked with severe organismal wounding that triggers processes like hemolymph coagulation and melanization and thereby alters hemolymph properties [68], [86], [87]. To limit melanization, we isolated hemolymph from *Bc* mutant larvae, in which the activity of phenoloxidase, a key enzyme for melanization, is absent from hemolymph [88]. Moreover, we developed a procedure for isolation of larval hemolymph [52] that provided us with quantities sufficient for our standard small scale disc culture set up. Unfortunately, the isolated hemolymph turned out to be highly toxic not only for isolated wing imaginal discs but also for S2R+ cells. Toxicity was observed with undiluted hemolymph as well as after dilution with Mcl8 medium (1∶2, 1∶4) (Fig. 8A) Cellular deterioration occurred considerably faster in hemolymph media compared to standard culture medium.

As isolated hemolymph was found to be unsuitable for wing imaginal disc culture, we explored whether the Mcl8 culture medium might be improved by addition of factors that have been recognized as important for long term culture in other systems. Generally, the function of various growth factors and mitogens is affected by the composition of basement membranes and extracellular matrix [89]–[91] and mammalian organoid development depends on matrigel, a solubilized basement membrane preparation [92]–[94]. Interestingly, in *Drosophila* larvae major components of the basement membrane of wing imaginal discs are delivered primarily by the fat body [95]. Isolated wing imaginal discs might therefore be unable to maintain basement membrane functionality in culture. Unfortunately, addition of matrigel, a basal membrane extract (BME) shown to be essential for mammalian organoid culture, did not prevent the rapid cell cycle arrest in cultured wing imaginal discs (Fig. 8A).

Among the known growth factors, insulin and insulin-like growth factors have been most extensively and most directly linked to growth stimulation in cultured cells and tissues [96]. The wing imaginal disc clone 8 cell line is routinely cultured in medium containing moderate levels of bovine insulin (50 µg/ml). However, high concentrations of bovine insulin (200 µg/ml) have been demonstrated to be crucial for cultivation of *Drosophila* ovaries and testis [97]–[99]. Recently, bovine insulin at low concentration (6.2 µg/ml) has been shown to be more effective when present in an improved medium for wing imaginal disc cultivation (WM1) compared to Mcl8 [30], but an analysis of the effects of high insulin concentrations has not yet been performed. Therefore, we analyzed the effects of supplementation of both Mcl8 and WM1 with high concentrations of bovine insulin (200 µg/ml). In both cases, addition of high levels of bovine insulin had some effect. The number of mitotic cells observed after 7 hours of wing imaginal disc cultivation was significantly increased after supplementation with high bovine insulin levels in comparison to standard Mcl8 and WM1 (Fig. 8A, B). Our experiments also confirmed that in comparison to Mcl8, WM1 is superior at keeping the number of mitotic cells high in cultured discs (without and with additional supplements). However, after 14 hours of cultivation the number of mitotic cells was low even in the presence of high bovine insulin (Fig. 8A, B). We conclude that high levels of bovine insulin delay but do not prevent the cell proliferation arrest during disc culture.

The concentration of bovine insulin that improves long term cultivation of ovaries [97], [98], testis [99] and wing imaginal discs (Fig. 8) is remarkably high. Bovine insulin might not be an optimal agonist of the *Drosophila* insulin receptor (Inr), and thus high concentrations might be required. Endogenous Inr ligands, the *Drosophila* Insulin-like peptides (Dilps) are encoded by several genes [12]. Some of these Dilps are secreted by dedicated neurosecretory cells located in the fly brain and, according to genetic analysis, Dilp2 is the most potent growth promoter among these [12]. Therefore, we analyzed the effect of medium supplementation with chemically synthesized Dilp2. Experiments with Kc cells clearly demonstrated that our Dilp2 preparation was active. Addition of Dilp2 increased the level of activated Akt1 (Akt1 phosphorylated on Serine 505) to a comparable extent as bovine insulin (Fig. 8C) [100]. We analyzed whether Dilp2 might support long term growth of wing imaginal discs in culture more effectively than bovine insulin. Our results demonstrate that moderate levels of Dilp2 (0.5 µM) are similarly effective as high levels of bovine insulin (35 µM, i.e., 200 µg/ml) in preventing a rapid cell proliferation arrest during disc culture (Fig. 8B). However, as also observed with bovine insulin, Dilp2 did not prevent the eventual cell proliferation arrest (Fig. 8B).

The fact that bovine insulin and Dilp2 were found to delay cell proliferation arrest during culture of wing imaginal discs suggested that an absence of essential endocrine factors in the medium might be responsible for the failure of long term growth and development of imaginal discs in culture. Accordingly, co-culture of discs with *Drosophila* larval tissues that produce such putative endocrine signals should be beneficial. In fact, earlier studies have indicated a beneficial effect of fat body co-culture on imaginal disc growth in *Drosophila* [32]. Similarly, growth of imaginal discs from the butterfly *Precis coenia* [37] and tobacco hornworm *Manduca sexta* [38] in culture is effectively stimulated by brain extract or hemolymph. Therefore we evaluated the effect of adding fat body tissue and whole brains from *Drosophila* third instar larvae to Mcl8 medium on cell proliferation in cultured wing imaginal discs. While these additions appeared to have some positive effect (p>0.05, t test), the number of mitotic cells after 7 hours of disc culture was nevertheless still very low (Fig. 8D).

### Cultivation of partially dissected wing imaginal discs delays cell proliferation arrest

Tissues other than brain and fat body might provide endocrine signals essential for growth and development of imaginal discs. Therefore we performed experiments with wing imaginal discs that were cultured together with the anterior larval thirds. For these experiments, wing imaginal discs were not completely dissected away from the larvae. This partial dissection (Fig. 8E, F) appeared attractive also because it results in reduced mechanical stress exerted on wing imaginal discs and co-cultured larval tissues during culture set-up. The preparation avoids damage to the physical connections between wing discs and surrounding larval tissue. Thus tracheal trunks and stalk cells, which entertain intimate physical interactions with the wing disc, were present in the cultures, as well as larval brains, fat body parts and other tissues present in the anterior thirds of the larvae. Interestingly, the number of mitotic cells observed in such partially dissected discs after 7 hours of cultivation was significantly higher compared with completely dissected control disc (Fig. 8F). With a number of additional experiments, we tried to clarify whether endocrine effects or integrity of physical disc attachment were responsible for the beneficial effects of partial dissection. Therefore, we co-cultured completely dissected discs with partially dissected discs (Fig. 8G). Moreover, we also started cultures where partial dissection was followed by complete dissection of the wing imaginal discs before culture onset. Finally, we prepared cultures with imaginal discs that still had their intimate physical associations with tracheal trunk and stalk cells but otherwise did not contain additional larval tissues. The analysis of the number of mitotic cells observed after 7 hours of cultivation in these experiments (Fig. 8G) suggested that endocrine effects and physical integrity might both contribute to the beneficial effect of partial disc dissection.

Finally, we analyzed whether the beneficial effects of partial disc dissection could be further enhanced by supplementation with high levels of bovine insulin (Fig. 8G). Indeed a further moderate increase in the number of mitotic cells detected after 7 hours of cultivation was observed when partial dissection and high bovine insulin were applied. After 14 hours of cultivation this additive effect was even more pronounced and significant (p = 0.002) compared to high bovine insulin after complete dissection. We conclude that a combination of partial dissection of wing imaginal discs and supplementation with high levels of bovine insulin is most effective in supporting cell proliferation during disc culture in vitro.

## Discussion

Our work on in vitro culture of *Drosophila* wing imaginal discs demonstrates the strikingly demanding nature of this tissue. Culture conditions that are fully adequate to support growth and proliferation of cultured *Drosophila* cells or of imaginal discs from lepidopteran species induce within few hours an unhealthy state in cells of *Drosophila* wing imaginal discs that is incompatible with cell proliferation. While we have not succeeded so far in overcoming all impediments, we describe significant methodological improvements that prolong cell proliferation in cultured discs substantially. Supplementation of culture medium with high levels of insulin is shown to be clearly beneficial. Moreover, we demonstrate that disc preparation by a partial dissection technique further contributes to sustained cell proliferation in culture.

Our transplantation experiments confirm that isolation of wing imaginal discs does not already cause irreversible damage that prevents subsequent cell growth and proliferation. We show that cell proliferation within transplanted discs proceeds in the abdomen of adult female hosts at a rate that is only twofold lower than during unperturbed discs growth situ. While growth of transplanted imaginal discs has been documented in many publications since the pioneering development of disc transplantation in *Drosophila* [101], [102], the temporal dynamics of cell proliferation has not yet been analyzed carefully. By counting cells (rather than just estimating overall disc size as in most earlier publications) and analyzing time points already soon after transplantation, we find that imaginal cell proliferation resumes without a substantial lag. Our time-resolved cell counts are also of interest with regard to disc-intrinsic cell proliferation control which has obtained major support by a previous widely cited study [7] where more mature wing discs were transplanted than in our experiments. These more mature discs were isolated just one cell population doubling before they had arrived at the terminal cell number reached during normal development in situ. After transplantation of such advanced discs, one cell population doubling was detected by counting cells at weekly intervals [7]. In our experiments, where considerably more immature discs were transplanted, cell numbers were observed to plateau rapidly after four exponential cell population doublings at about half of the target cell number reached in situ. Therefore, our results considerably strengthen the notion that cell proliferation in transplanted wing imaginal discs is determinate, although proliferation arrest might not occur precisely at the normal in situ target cell number.

Our larval wounding experiments also support the conclusion that disc dissection does not necessarily cause damage and consequential failure of sustained growth and development in vitro. We observe that larval wounding does not lead to a rapid block of mitosis within imaginal discs. Unfortunately, however, larval wounding transforms hemolymph eventually into a toxic medium, as suggested by our unexpected findings with discs cultured in isolated hemolymph. It is quite inconceivable that induction of toxicity in hemolymph as an immediate response to larval wounding might have evolved as such a reaction would hamper organismal recovery. The known rapid and prominent wounding responses in hemolymph (coagulation, melanization) are thought to support healing and, analogous to very well established observations from mammalian organisms, are far more likely to generate growth stimulating and mitogenic activities rather than the opposite. Thus hemolymph toxicity might develop secondarily during prolonged maintenance of this fluid outside of the larval body. Toxicity might develop during prolonged exposure to the non-physiologically high atmospheric concentrations of oxygen or as a result of an absence of detoxifying regenerative activities of certain larval tissues. An identification of the toxic constituents in isolated hemolymph will not be trivial as its composition is very complex [52].

Conventional culture media based on Shields and Sang's M3 or Schneider's medium are less damaging for discs in culture than isolated hemolymph. But also these media are unable to keep imaginal disc cells in a healthy state. EdU and anti PH3 labeling, as well as our novel RGB cell cycle reporter system all indicate that cell cycle progression rapidly ceases in cultured discs. The arrest does not occur at a specific cell cycle checkpoint, and forced expression of E2f1/Dp, a regulator limiting for progression through both the G1/S and the G2/M transition in imaginal discs growing in situ [40], cannot overcome cell proliferation arrest in culture. Disc culture also appears to affect biological processes beyond growth and proliferation. Our analysis of inducibility of *UAS-EGFP* expression via GAL80^ts^/GAL4 suggests that gene expression is severely compromised in cultured discs. Suppression of the JNK pathway, which is known to be activated by various types of stress, by overexpression of the negative feedback regulator puckered, did not appear to affect the deterioration of disc cells during culture. As indicated by our co-culture experiments, this disc cell deterioration during culture proceeds in conditions that do not interfere with proliferation of S2R+ cells. Clearly, imaginal disc cells have some specific dependencies that are irrelevant for proliferation of immortalized *Drosophila* cell lines.

The molecular basis of immortalization that has allowed establishment of S2R+ cells and other *Drosophila* cell lines is unknown. However, immortalization of human and rodent cells frequently involves mutational alterations in signaling pathways controlling growth and proliferation. Compared to primary cells, immortalized cells are often less dependent on growth factors and mitogens. In *Drosophila* wing imaginal discs, insulin receptor, Ras and Yki signaling have all been demonstrated to promote growth and proliferation [78], [79], [85], [103]. By expressing activated forms of Ras and Yki just before onset of disc culture, we were unable to overcome cell proliferation arrest in vitro. These results argue that the failure of sustained growth and proliferation of wing imaginal discs is unlikely a result of a loss of upstream activation of these pathways upon cultivation. In contrast, bovine insulin as well as Dilp2 was found to be effective in delaying cell proliferation arrest in cultured discs. However, also repeated addition of Dilps was found to be unable to prevent an eventual premature cell proliferation arrest. Thus sustained growth and proliferation of wing imaginal discs in culture presumably depends on factors other than Dilps.

Co-culture of wing imaginal discs with other larval tissues can potentially provide the missing unknown factors crucial for sustained disc growth and proliferation. Co-culture with both brains and fat bodies together appeared to delay cell proliferation arrest in cultured wing imaginal discs slightly in our experiments (but not with statistical significance). Moreover, culture of wing imaginal discs after isolation by a partial dissection which results in co-culture with the anterior third of the larva was found to result in a statistically significant delay in the cell proliferation arrest. These observations encourage a further evaluation of co-culture approaches. We add that the partial dissection procedure appears to be beneficial also because it minimizes mechanical stress during disc preparation.

By combining supplementation with high levels of insulin and partial dissection we have been able to develop conditions that delay the cell proliferation arrest in cultured wing imaginal discs strongly. This approach should therefore be very helpful in minimizing artefacts during short term analyses. In addition, our systematic analyses will hopefully accelerate future progress towards culture conditions that are fully compatible with long term development of wing imaginal discs.

## Supporting Information

Figure S1 An *RGB* cell cycle tracker reveals cell cycle progression of S2R+ cells. Time lapse in vivo imaging with S2R+ cells stably transfected with the pUbi-*RGB* cell cycle tracker. A single transcript expressed from the *RGB* cell cycle tracker construct results in production of three distinct proteins as a result of intervening T2A cis-acting hydrolase elements: (1) nlsCdt1^1–101^-EBFP2 (Blue), a blue fluorescent nuclear protein degraded during S phase because of the Cdt1 degron, (2) nlsCycB^1–96^-nlsCycB^1–285^-tdTomato (Red), a red nuclear protein degraded during late M and G1 because of Cyclin B degrons, and (3) EGFP-PCNA (Green), a green nuclear protein with a characteristic distinct subnuclear pattern during S phase. Still frames illustrate progression of a representative cell through the cell cycle. Cell cycle progression is accompanied by characteristic rapid changes in the expressed color combination and subnuclear GFP pattern at cell cycle transitions. Time (hours∶minutes) after onset of time lapse imaging is indicated below the still frames. Scale bar = 5 µm.
(TIF)

Figure S2 Effect of *puc* overexpression on cell proliferation during imaginal wing disc culture. Imaginal wing discs were isolated from *Lac-YFP* larvae with *en-GAL4* driving expression of either only *UAS-mCherry-nls* or *UAS-mCherry-nls* in combination with *UAS-puc*. After isolation at 100 hours AED, discs were cultured in vitro and analyzed by time lapse imaging during the indicated time periods. The number of mitotic divisions during these monitored time intervals were determined in the anterior (A) and posterior (P) disc compartment within a reference area of 6800 µm^2^. For each genotype, at least 10 discs were analyzed. Bars indicate average number of mitotic cells +/− s.d.
(TIF)

Figure S3 Effect of larval wounding on the number of mitotic cells in wing imaginal discs. Third instar larvae (100 hours AED) were wounded by either single (squares) or multiple (three times, triangles) penetrations of the larval cuticle at the posterior end with a fine glass needle. While single penetration had only a minor effect on survival of the treated larvae (90%), multiple penetrations led to a clear reduction (60%). At various time points after wounding, wing imaginal discs were dissected and fixed immediately for analysis of the number of mitotic cells. (average +/− s.d., n≥9). The number of mitotic cells observed in wing imaginal discs from mock-treated larvae at 0 hours, which was 51 (+/−8.0 s.d., n = 19), was set as 100%.
(TIF)

Figure S4 Effect of wing imaginal disc co-culture on S2R+ cell proliferation. (A, B) Twenty four hours after plating of S2R+ cells in Schneider's medium, wing imaginal discs were either added to the cultures (+ discs) or not (- discs). At the time of disc addition, Schneider's medium was exchanged with Mcl8 in the cultures. (A) S2R+ cell density was monitored by phase contrast microscopy at identical positions at the time of disc addition and 48 hours later. Scale bar  = 50 µm. (B) 24 and 48 hours after the time of disc addition, S2R+ cells cultured with or without discs were pulse labeled with EdU followed by anti-PH3 and DNA staining. The fraction of S2R+ cells in M phase (PH3 positive) and S phase (EdU positive) were determined. The results of two independent experiments are displayed on top of each other.
(TIF)

Figure S5 Effect of oxygen level on the number of mitotic cells in cultured wing imaginal discs. (A, B) Wing imaginal discs expressing EGFP-ODD and nls-mRFP from transgenes under control of the *pUbi-p63E* cis-regulatory region were cultured in vitro for 7 hours in an atmosphere with 5%, 21% or 60% oxygen. ODD, the oxygen-dependent degradation domain of Sima, the *Drosophila* homolog of Hypoxia inducible factor-1 alpha, confers oxygen-dependent stability regulation on EGFP. (A) The number of mitotic cells was found to be maximal after culture at 21% oxygen. Bars indicate average number of mitotic cells +/− s.d. (B) Relative to nls-RFP signals, EGFP-ODD signals were inversely correlated with oxygen levels, as expected. Average signals observed with 5% oxygen were set to 1 arbitrary unit (a.u.). and determined the number of mitotic cells after 7 hours of culture (Fig. S5).
(TIF)

Figure S6 Temporally and spatially controlled Ras85D^V12^ expression in wing imaginal discs. (A, B) *en-GAL4 tub-GAL80^ts^ UAS-Ras85D^V12^* larvae were used for hyperactivation of the Ras signaling pathway in the posterior compartment of wing imaginal discs by temperature shift. Wing imaginal discs from larvae that had been kept constantly at 23°C (A) or from larvae that had been shifted to 29°C for the final 12 hours before disc isolation (B) were fixed either before (0 h) or after seven hours of cultivation (7 h) and labeled with anti-Ras and a DNA stain. In (B), anti-Ras signals were slightly but clearly elevated in the posterior compartment. Scale bar = 50 µm.
(TIF)

Figure S7 Effects of Yki^3A^ expression on cell cycle progression during wing imaginal disc cultivation. (A) *en-GAL4 tub-GAL80^ts^ UAS-Yki^3A^* larvae were used for spatially and temporally controlled hyperactivation of the Yorkie pathway as illustrated schematically. (B-E) Wing imaginal discs dissected from larvae constantly grown at 23°C (B, D) or from larvae shifted to 29°C for the final 12 hours before disc isolation (C, E) were fixed either immediately (0 h) or after seven hours of cultivation in vitro (7 h) before labelling with EdU and staining with anti-PH3 and a DNA dye. Complete imaginal discs (B, C) and high magnification views of the central pouch region (D, E) are shown with dashed lines indicating the boundary between anterior and posterior compartment, in which *en-GAL4* driven *UAS* transgene expression occurs at 29°C. There were clearly more EdU- and PH3-positive cells in the posterior compartment of wing imaginal discs analyzed immediately after dissection from larvae that had been exposed to 29°C (C, E, 0 h). However, after seven hours of disc cultivation, EdU incorporation and the number of PH3-positive cells was strongly reduced to a comparable level in both the anterior and the posterior compartment in discs with and without *Yki^3A^* expression in the posterior compartment before disc isolation and cultivation (B-E, 7 h). Scale bar corresponds to 50 µm (B, C) and 20 µm (D, E), respectively.
(TIF)
