# Supplementary figures and images for: Towards Long Term Cultivation of Drosophila Wing Imaginal Discs In Vitro
Source: PLoS One. 2014 Sep 9;9(9):e107333. doi: 10.1371/journal.pone.0107333 (PMC4159298; doi:10.1371/journal.pone.0107333)

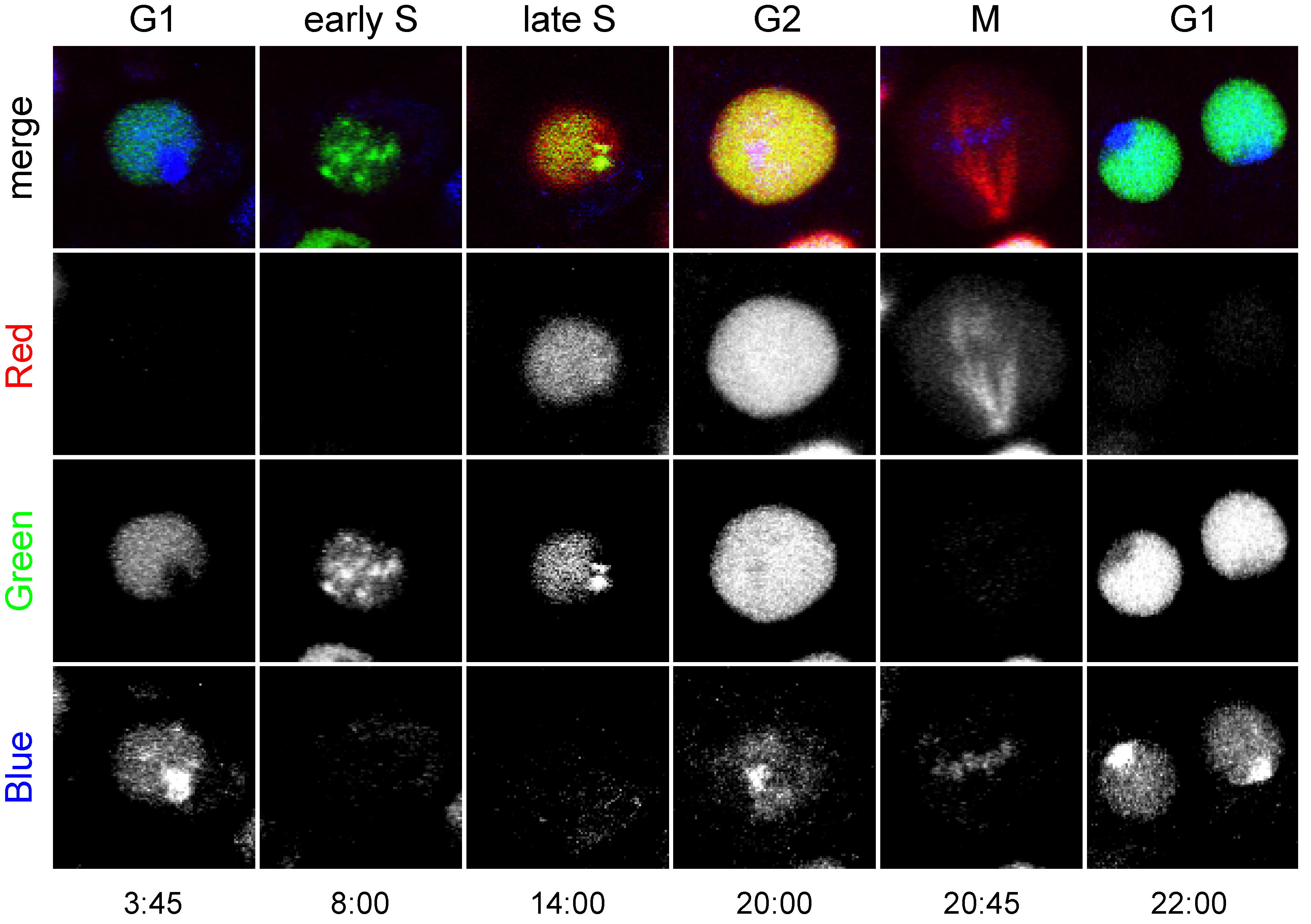

Supplement: Figure S1 — An RGB cell cycle tracker reveals cell cycle progression of S2R+ cells. Time lapse in vivo imaging with S2R+ cells stably transfected with the pUbi-RGB cell cycle tracker. A single transcript expressed from the RGB cell cycle tracker construct results in production of three distinct proteins as a result of intervening T2A cis-acting hydrolase elements: (1) nlsCdt11–101-EBFP2 (Blue), a blue fluorescent nuclear protein degraded during S phase because of the Cdt1 degron, (2) nlsCycB1–96-nlsCycB1–285-tdTomato (Red), a red nuclear protein degraded during late M and G1 because of Cyclin B degrons, and (3) EGFP-PCNA (Green), a green nuclear protein with a characteristic distinct subnuclear pattern during S phase. Still frames illustrate progression of a representative cell through the cell cycle. Cell cycle progression is accompanied by characteristic rapid changes in the expressed color combination and subnuclear GFP pattern at cell cycle transitions. Time (hours∶minutes) after onset of time lapse imaging is indicated below the still frames. Scale bar = 5 µm. (TIF) [file pone.0107333.s001.tif]

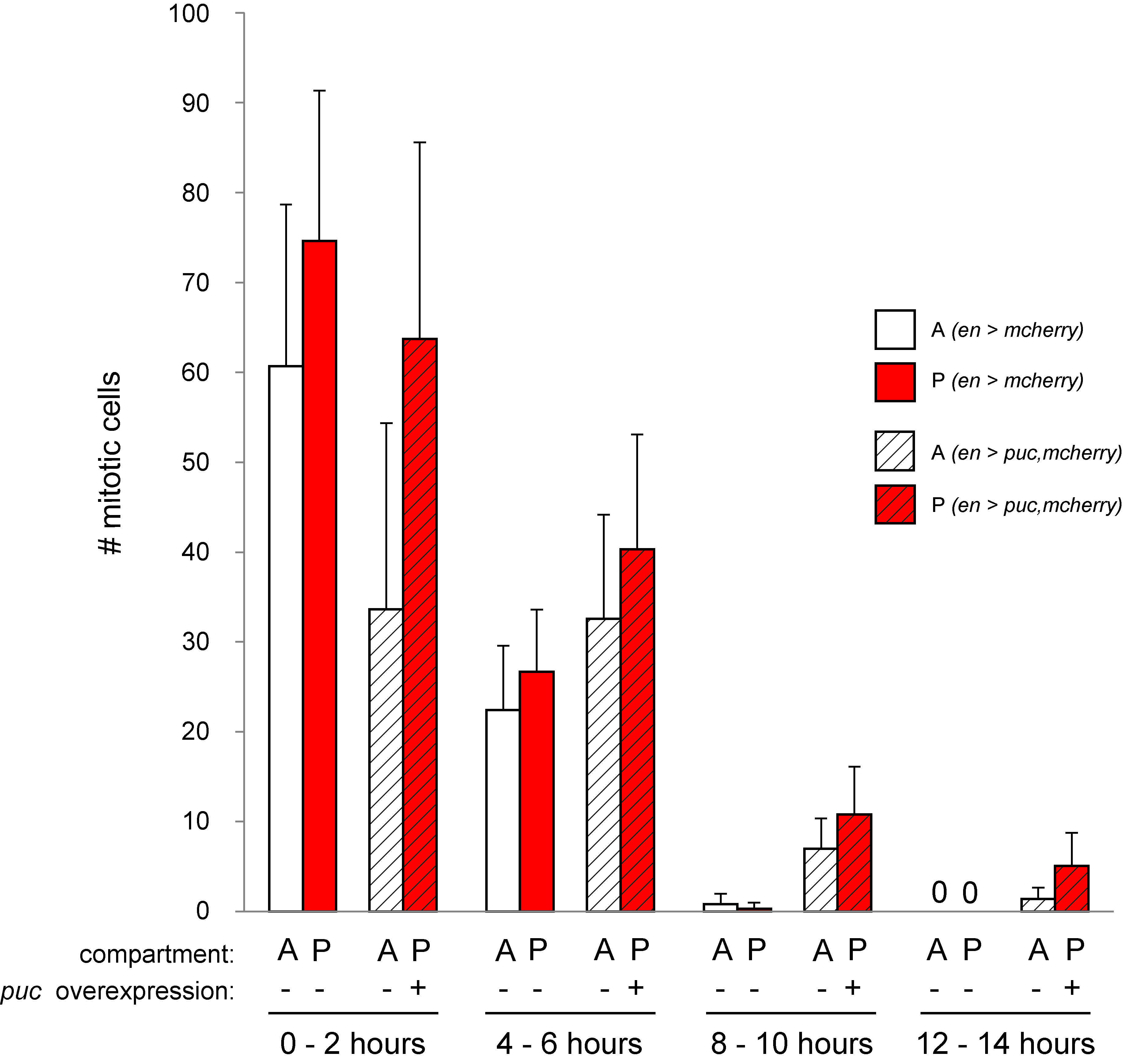

Supplement: Figure S2 — Effect of puc overexpression on cell proliferation during imaginal wing disc culture. Imaginal wing discs were isolated from Lac-YFP larvae with en-GAL4 driving expression of either only UAS-mCherry-nls or UAS-mCherry-nls in combination with UAS-puc. After isolation at 100 hours AED, discs were cultured in vitro and analyzed by time lapse imaging during the indicated time periods. The number of mitotic divisions during these monitored time intervals were determined in the anterior (A) and posterior (P) disc compartment within a reference area of 6800 µm2. For each genotype, at least 10 discs were analyzed. Bars indicate average number of mitotic cells +/− s.d. (TIF) [file pone.0107333.s002.tif]

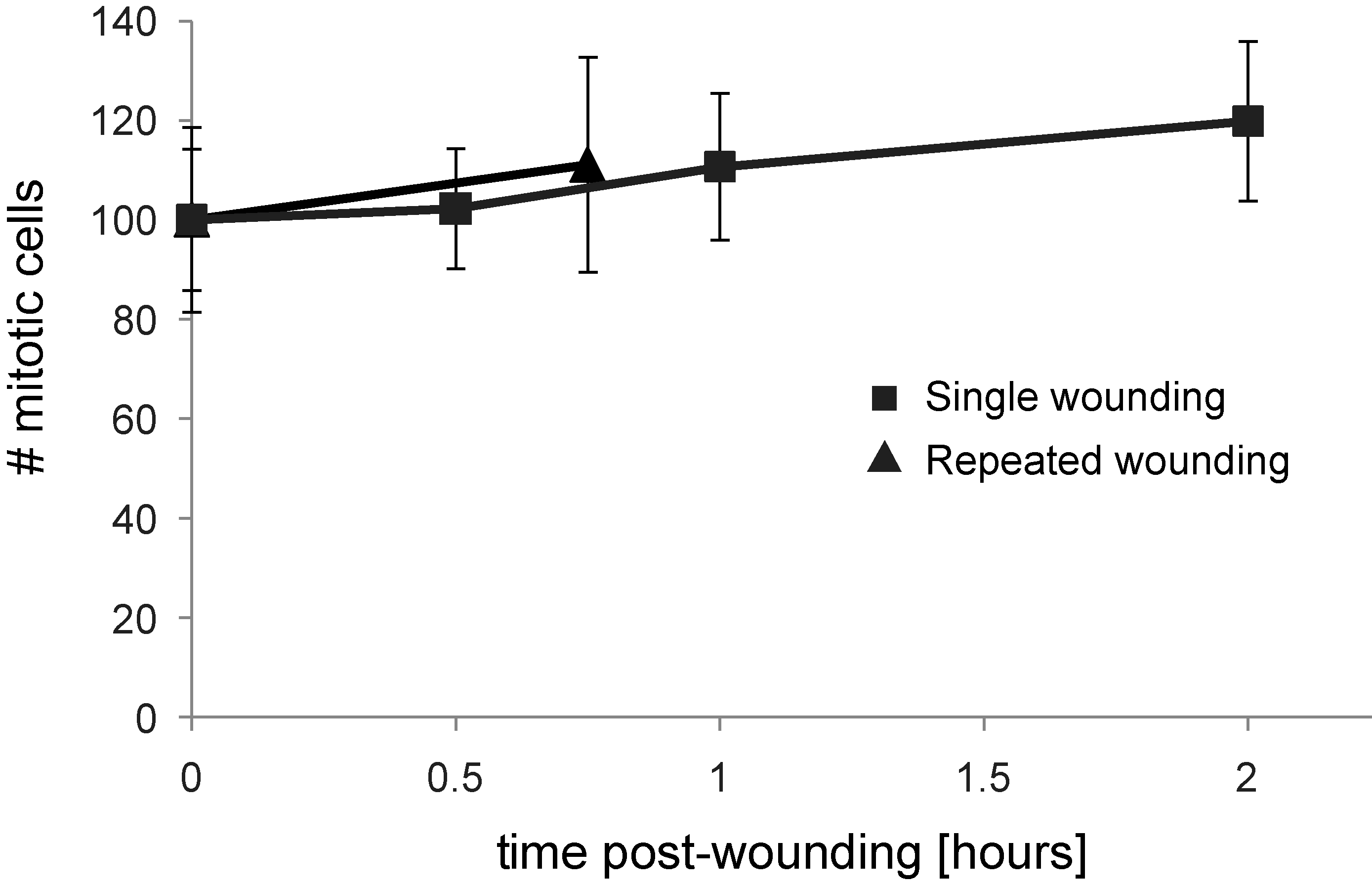

Supplement: Figure S3 — Effect of larval wounding on the number of mitotic cells in wing imaginal discs. Third instar larvae (100 hours AED) were wounded by either single (squares) or multiple (three times, triangles) penetrations of the larval cuticle at the posterior end with a fine glass needle. While single penetration had only a minor effect on survival of the treated larvae (90%), multiple penetrations led to a clear reduction (60%). At various time points after wounding, wing imaginal discs were dissected and fixed immediately for analysis of the number of mitotic cells. (average +/− s.d., n≥9). The number of mitotic cells observed in wing imaginal discs from mock-treated larvae at 0 hours, which was 51 (+/−8.0 s.d., n = 19), was set as 100%. (TIF) [file pone.0107333.s003.tif]

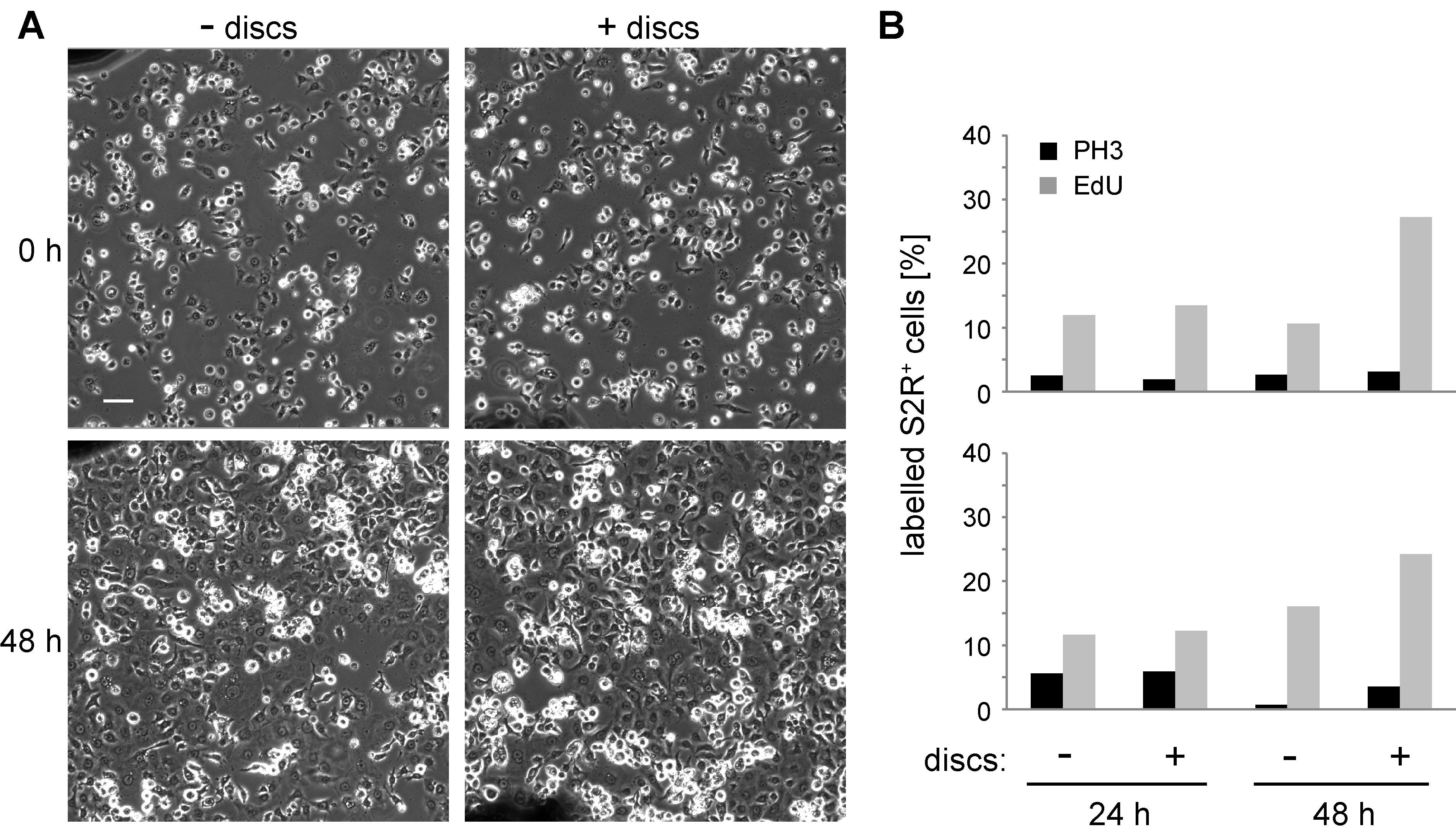

Supplement: Figure S4 — Effect of wing imaginal disc co-culture on S2R+ cell proliferation. (A, B) Twenty four hours after plating of S2R+ cells in Schneider's medium, wing imaginal discs were either added to the cultures (+ discs) or not (- discs). At the time of disc addition, Schneider's medium was exchanged with Mcl8 in the cultures. (A) S2R+ cell density was monitored by phase contrast microscopy at identical positions at the time of disc addition and 48 hours later. Scale bar = 50 µm. (B) 24 and 48 hours after the time of disc addition, S2R+ cells cultured with or without discs were pulse labeled with EdU followed by anti-PH3 and DNA staining. The fraction of S2R+ cells in M phase (PH3 positive) and S phase (EdU positive) were determined. The results of two independent experiments are displayed on top of each other. (TIF) [file pone.0107333.s004.tif]

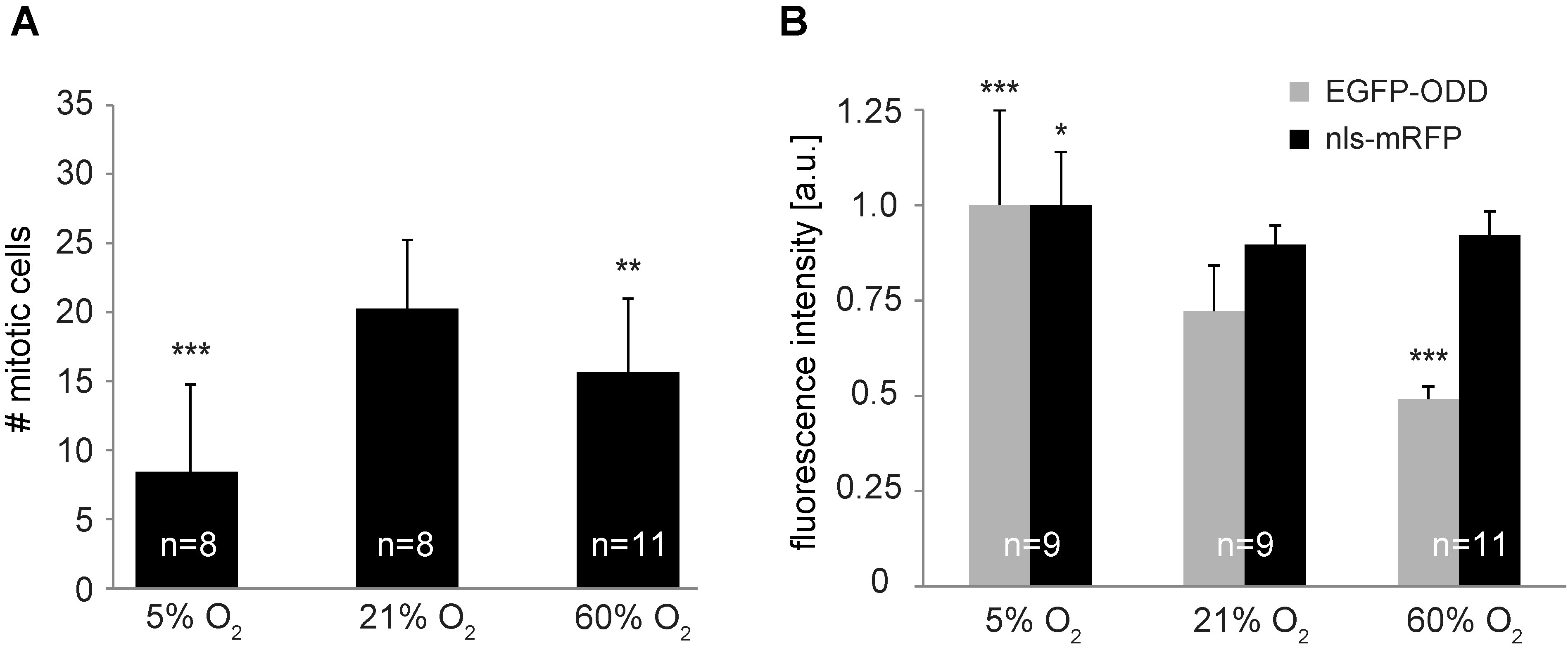

Supplement: Figure S5 — Effect of oxygen level on the number of mitotic cells in cultured wing imaginal discs. (A, B) Wing imaginal discs expressing EGFP-ODD and nls-mRFP from transgenes under control of the pUbi-p63E cis-regulatory region were cultured in vitro for 7 hours in an atmosphere with 5%, 21% or 60% oxygen. ODD, the oxygen-dependent degradation domain of Sima, the Drosophila homolog of Hypoxia inducible factor-1 alpha, confers oxygen-dependent stability regulation on EGFP. (A) The number of mitotic cells was found to be maximal after culture at 21% oxygen. Bars indicate average number of mitotic cells +/− s.d. (B) Relative to nls-RFP signals, EGFP-ODD signals were inversely correlated with oxygen levels, as expected. Average signals observed with 5% oxygen were set to 1 arbitrary unit (a.u.). and determined the number of mitotic cells after 7 hours of culture (Fig. S5). (TIF) [file pone.0107333.s005.tif]

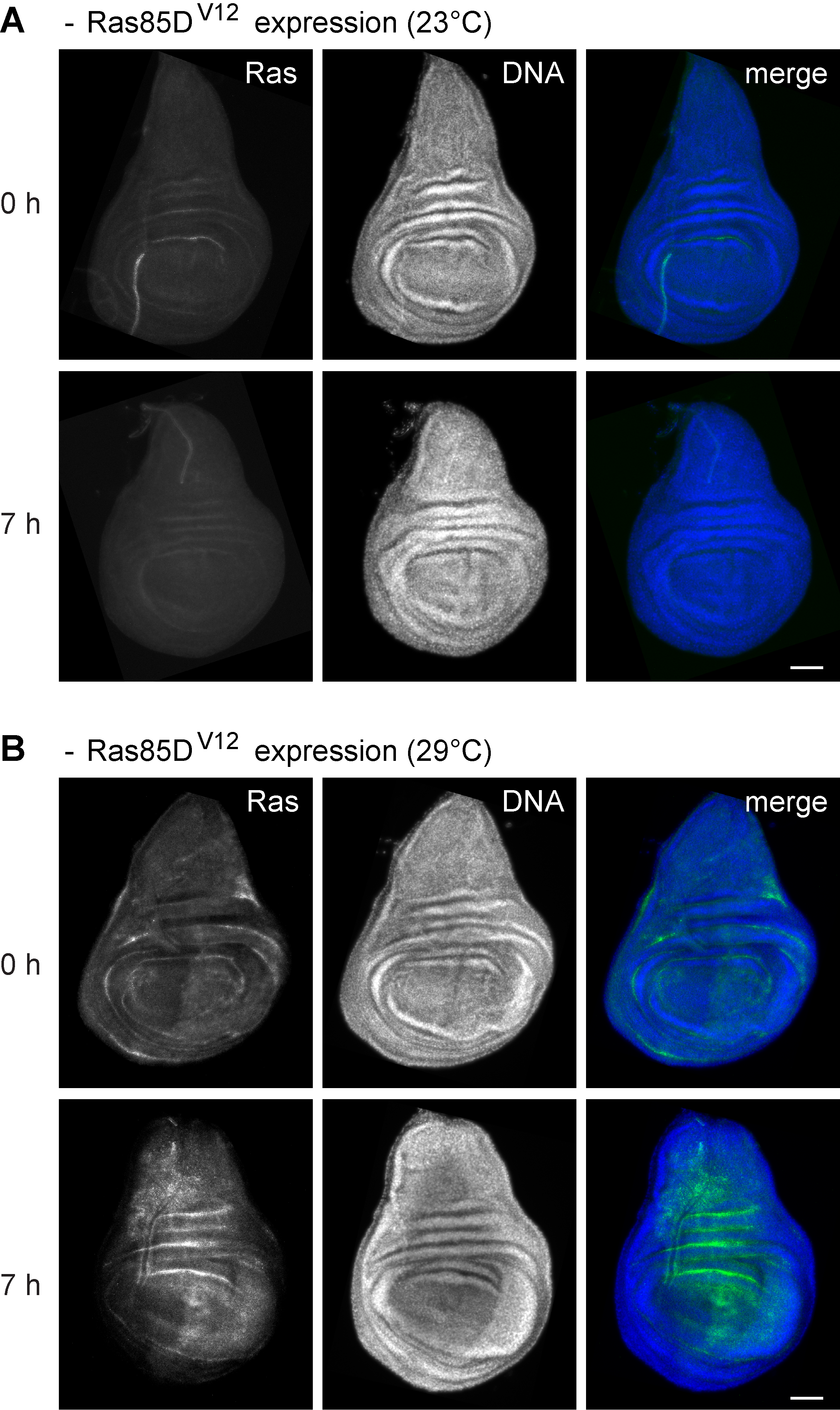

Supplement: Figure S6 — Temporally and spatially controlled Ras85DV12 expression in wing imaginal discs. (A, B) en-GAL4 tub-GAL80ts UAS-Ras85DV12 larvae were used for hyperactivation of the Ras signaling pathway in the posterior compartment of wing imaginal discs by temperature shift. Wing imaginal discs from larvae that had been kept constantly at 23°C (A) or from larvae that had been shifted to 29°C for the final 12 hours before disc isolation (B) were fixed either before (0 h) or after seven hours of cultivation (7 h) and labeled with anti-Ras and a DNA stain. In (B), anti-Ras signals were slightly but clearly elevated in the posterior compartment. Scale bar = 50 µm. (TIF) [file pone.0107333.s006.tif]

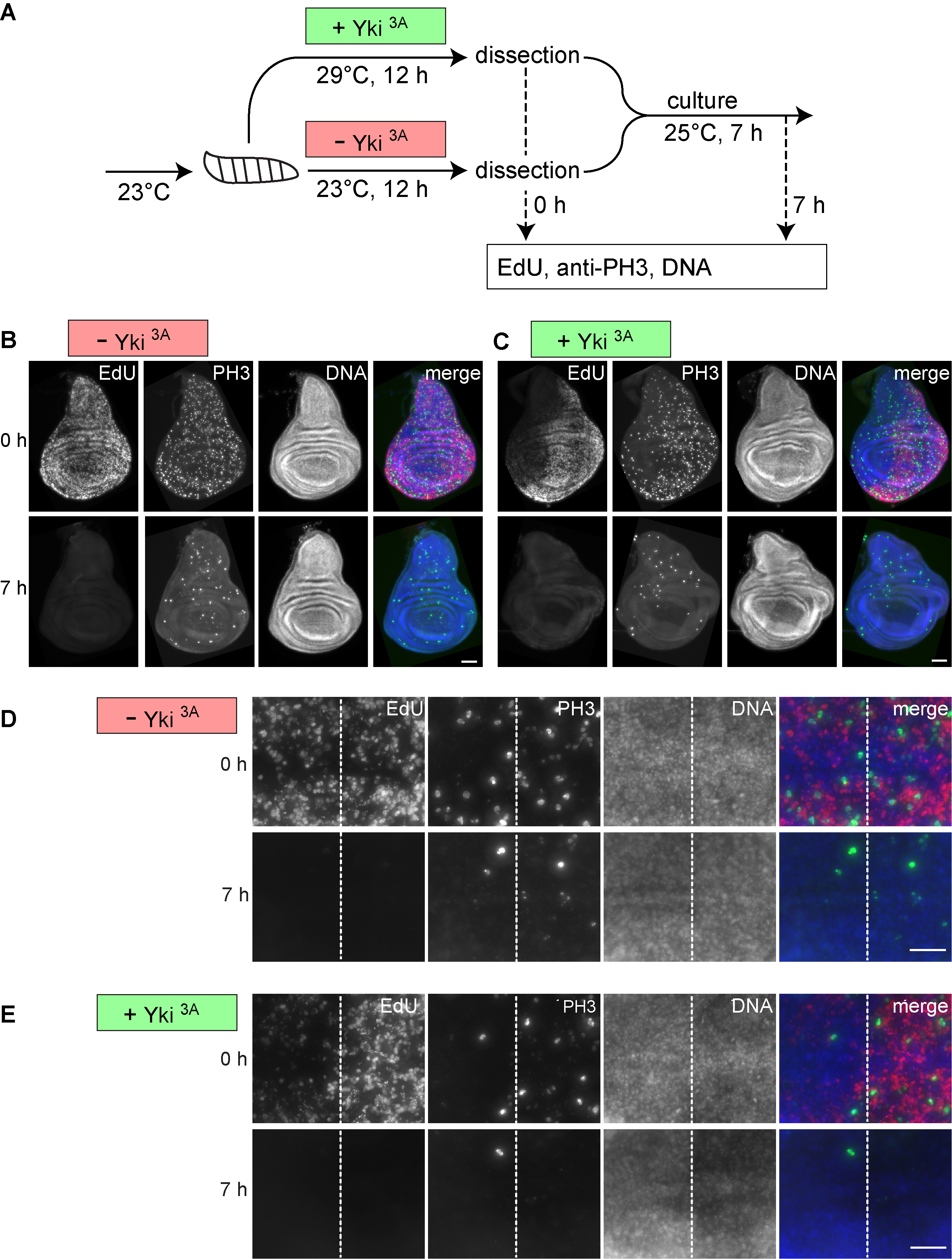

Supplement: Figure S7 — Effects of Yki3A expression on cell cycle progression during wing imaginal disc cultivation. (A) en-GAL4 tub-GAL80ts UAS-Yki3A larvae were used for spatially and temporally controlled hyperactivation of the Yorkie pathway as illustrated schematically. (B-E) Wing imaginal discs dissected from larvae constantly grown at 23°C (B, D) or from larvae shifted to 29°C for the final 12 hours before disc isolation (C, E) were fixed either immediately (0 h) or after seven hours of cultivation in vitro (7 h) before labelling with EdU and staining with anti-PH3 and a DNA dye. Complete imaginal discs (B, C) and high magnification views of the central pouch region (D, E) are shown with dashed lines indicating the boundary between anterior and posterior compartment, in which en-GAL4 driven UAS transgene expression occurs at 29°C. There were clearly more EdU- and PH3-positive cells in the posterior compartment of wing imaginal discs analyzed immediately after dissection from larvae that had been exposed to 29°C (C, E, 0 h). However, after seven hours of disc cultivation, EdU incorporation and the number of PH3-positive cells was strongly reduced to a comparable level in both the anterior and the posterior compartment in discs with and without Yki3A expression in the posterior compartment before disc isolation and cultivation (B-E, 7 h). Scale bar corresponds to 50 µm (B, C) and 20 µm (D, E), respectively. (TIF) [file pone.0107333.s007.tif]
